# Impact of Chronic Exposure to Two Neonicotinoids on Honey Bee Antennal Responses to Flower Volatiles and Pheromonal Compounds

**DOI:** 10.3389/finsc.2022.821145

**Published:** 2022-04-18

**Authors:** Riccardo Favaro, Jacob Roved, Albrecht Haase, Sergio Angeli

**Affiliations:** ^1^Faculty of Science and Technology, Free University of Bozen-Bolzano, Bolzano, Italy; ^2^Section for Evolutionary Genomics, Faculty of Health and Medical Sciences, GLOBE Institute, University of Copenhagen, Copenhagen, Denmark; ^3^Center for Mind/Brain Science (CIMeC), University of Trento, Rovereto, Italy; ^4^Department of Physics, University of Trento, Povo, Italy

**Keywords:** honey bee, pesticides, neonicotinoids, electroantennography, olfaction, brain, queen mandibular pheromone, alarm pheromone

## Abstract

Volatile compounds provide important olfactory cues for honey bees (*Apis mellifera* L.), which are essential for their ecology, behavior, and social communication. In the external environment bees locate food sources by the use of floral scents, while inside the hive, pheromones such as the queen mandibular pheromone (QMP) and alarm pheromones serve important functions in regulating colony life and inducing aggressive responses against intruders and parasites. Widely reported alterations of various behaviors in- and outside the hive following exposure to pesticides could therefore be associated with a disturbance of odor sensitivity. In the present study, we tested the effects of neonicotinoid pesticides at field concentrations on the ability of honey bees to perceive volatiles at the very periphery of the olfactory system. Bee colonies were subjected to treatments during the summer with either Imidacloprid or Thiacloprid at sublethal concentrations. Antennal responses to apple (*Malus domestica* L.) flower volatiles were studied by GC-coupled electro-antennographic detection (GC-EAD), and a range of volatiles, a substitute of the QMP, and the alarm pheromone 2-heptanone were tested by electroantennography (EAG). Short-term and long-term effects of the neonicotinoid treatments were investigated on bees collected in the autumn and again in the following spring. Treatment with Thiacloprid induced changes in antennal responses to specific flower VOCs, with differing short- and long-term effects. In the short term, increased antennal responses were observed for benzyl-alcohol and 1-hexanol, which are common flower volatiles but also constituents of the honey bee sting gland secretions. The treatment with Thiacloprid also affected antennal responses to the QMP and the mandibular alarm pheromone 2-heptanone. In the short term, a faster signal degeneration of the response signal to the positive control citral was recorded in the antennae of bees exposed to Thiacloprid or Imidacloprid. Finally, we observed season-related differences in the antennal responses to multiple VOCs. Altogether, our results suggest that volatile-specific alterations of antennal responses may contribute to explaining several behavioral changes previously observed in neonicotinoid-exposed bees. Treatment effects were generally more prominent in the short term, suggesting that adverse effects of neonicotinoid exposure may not persist across generations.

## Introduction

Recent research has demonstrated a strong decline of various groups of pollinating insects, although future prospects of crop production rely on an increased dependency of agriculture on pollination services (1). Honey bee species are essential for their pollination services, contributing to the conservation of wild plant biodiversity as well as to agricultural productivity (2). The Western honey bee, *Apis mellifera* L., is the most important managed pollinator worldwide and a primary species for pollination of agricultural crops (3). In an effort to contrast raising concerns about current and future capacities of pollination service and guarantee food security, the EU parliament and EU commission have recently launched the “Farm to fork” and “Green Deal” initiatives, imposing a 50% reduction in pesticide use and an increase by 25% of organic farming by the year 2030 (4). It is therefore necessary to develop more bee-friendly agricultural practices and support farmers with reorganization toward less intensive industrial agriculture (5).

Despite extensive research, there is no conclusive evidence as to the cause of the massive decline of managed honey bee colonies registered during recent years in Europe and the USA. While multiple interacting factors such as pesticides exposure, the impact of the parasitic mite *Varroa destructor*, agricultural intensification, and climate change may play a role (6), the use of pesticides in large agricultural landscapes poses a serious threat to bee health and colony survival. In a recent study on bees collected along an alpine valley (7), 63 agrochemicals were found, 15 insecticides, 43 fungicides, 3 herbicides and 2 plant hormones. Furthermore, the drift of compounds such as Phosmet and Fluazinam have been shown to pose serious risks to the environment and to human health as far as 10 km away from treated areas (7).

Honey bees may get exposed to pesticides either through topical contact or indirectly through consumption of contaminated nectar, pollen, or water (e.g., guttation water) (8). Traditionally, the measurement of toxic effects of pesticides has relied largely on the determination of acute toxicity, while chronic and sublethal effects have received less attention. However, prolonged use of a large, diverse set of pesticides in agricultural landscapes may have detrimental effects on the health of bees and threaten the survival of bee colonies long after pesticide use. Whilst acute exposure results in rapid appearance of symptoms, the consequences following a chronic exposure are harder to assess, partly because bees degrade pesticides through a continuous food processing and thereby reduce the amount of pesticide residuals in the hive matrix (9).

A key aspect for the survival of bees is their ability to forage, orientate, and return home with collected resources such as nectar, pollen and water. Flower scents play an important role in bee orientation, allowing them to recognize and locate specific plant species during foraging flights, which is essential to the pollination service provided by bees (10, 11). In addition, pollination efficiency is also affected by the ability to detect pollen and nectar *via* odor cues inside flowers (10, 12). Potential changes in the detection of flower volatiles may cause disturbance in the foraging behavior of bees, with implications on both bee survival and pollination activity. As a social insect, honey bees also rely strongly on pheromonal communication, e.g., with the release and perception of the queen mandibular pheromone (QMP) and the brood pheromone within the hive, or the alarm pheromone (e.g., 2-heptanone) and the aggregation pheromone outside the hive (13, 14). Pheromones have also been found to affect motivation, learning, and memory of bees (15).

Neonicotinoids are commonly used as insecticides and previous studies have demonstrated that bee colonies chronically exposed to neonicotinoids have a reduced number of adult bees (−28%), brood surface (−13%), and pollen collection (−19%), and tend to exhibit higher queen supersedure as a long-term impact (16). Physiological studies have demonstrated that neonicotinoids interfere with the neural signal transduction as an agonist of insect nicotinic acetylcholine receptors at the postsynaptic membrane (17). Thus, neonicotinoids affect transmission from olfactory receptor neurons *via* the antennal lobe (18, 19) to the mushroom bodies. However, these studies were all conducted downstream of the olfactory receptor neurons (ORNs) and leave open the question to what extent the observed effects originate in the ORNs or even in perireceptor events (20).

In the present study, we exposed honey bee colonies to sublethal dietary concentrations of two neonicotinoids, Imidacloprid and Thiacloprid, and examined short- and long-term effects on the olfactory responses of adult bees. Specifically, we investigated whether chronic exposure to Imidacloprid and Thiacloprid (i.e., administered over several weeks) affected honey bee antennal sensitivity by measuring electroantennogram responses to flower volatiles, an alarm pheromone, and a substitute of the queen mandibular pheromone.

## Materials and Methods

### Apple Flower Volatiles Collection

Flower volatile organic compounds (VOCs) were collected in April 2018 from five specimens of 8 years old apple trees (*Malus x domestica*, Borkhausen, cv Fuji grafted on M9 rootstock), located in an organically managed parcel of the Laimburg Research Center (Bolzano, Italy). The volatiles were collected on the day after the opening of all the flowers in the cluster. A shoot portion containing two flower clusters was enclosed in a plastic bag (BVOC-bag, Cuki oven bag, Bolzano, Italy) and air samples were collected using an adsorbent trap (glass tube, 6.5 × 0.55 × 0.26 cm, loaded with 1.5 mg activated charcoal; CLSA filter LR-type; Brechbühler AG, Schlieren, Switzerland), as described by Giacomuzzi et al. (21). Samples were collected daily from 11:00 to 14:00. The collected VOC samples were eluted from the adsorbent traps with 100 μl GC grade dichloromethane in GC glass vials (Sigma-Aldrich, Milan, Italy) and stored at −80°C prior to the GC-MS analysis. VOCs were collected also from an empty sampling bag as a negative control.

### Honey Bee Colonies

The experiment was conducted using nine honey bee [*Apis mellifera* ssp. carnica (Pollmann)] colonies from the experimental apiary of the Free University of Bolzano in Altenburg (Bolzano, Italy) during the summer 2018 and spring 2019. The colonies were located in a mixed forest (*Quercus pubescens* Willd., *Fagus sylvatica* L., *Pinus sylvestris* L., *Ostrya carpinifolia* Scop.), with crop fields (vineyards and apples) located in the lower valley at ca 1 km flight distance. The colonies were created in May 2018 by the shook swarm method from healthy colonies, managed according to good beekeeping practice, that had undergone regular sanitary treatments against the parasitic mite *Varroa destructor* (Anderson and Trueman). Swarms of 1.5 kg of adult bees were transferred into standard 10-frames Dadant-Blatt beehives for nomadic beekeeping with organic wax foundation (Il Pungiglione Soc. Coop, Italy) and provided with new sister-queens. The colonies were sorted into three treatment groups, each placed at a distance of 10 m from each other. After 5 days, they were treated with 50 ml of oxalic acid dihydrate sucrose solution (5% w/v) trickled in-between frames for control of *Varroa destructor*. All queens were accepted and the development of the colonies was assessed weekly. To sustain development and wax construction, each colony was fed for 6 weeks with 1 L of sugar syrup (Apiinvert, Südzucker AG, Germany). In October 2018, when there was no more brood, an oxalic acid treatment using the sublimation method was applied against *Varroa destructor*. No other mite treatment was carried out in the spring of 2019 before the EAG trials.

### Neonicotinoid Treatments

The pesticide treatments were carried out starting on July 17, 2018. That period corresponds to the end of the last main blossom in the area (chestnut, *Castanea sativa* L.), and it is also the moment when local beekeepers collect the honey suppers and begin to provide supplementary syrup to sustain strong colonies. Hence, the nectar income from the surrounding area was expected to be limited. At the beginning of the treatment, the brood was spread over at least four frames in each beehive. Each colony was provided twice a week with 500 ml sugar syrup with or without pesticides (Apiinvert, Südzucker AG, Germany) through a rapid top feeder for 6 weeks. Treatment syrups were freshly prepared and administered in the evening and all colonies consumed the syrup until the following morning. At the end of the 6-weeks treatment, no other feed supplements were given until the end of the experiment. Colonies did not suffer detectable losses nor collapsed, and all reached the autumn season in good condition. All colonies survived the winter and successfully developed in spring 2019.

Insecticides were previously dissolved in acetone (>99.8%, Sigma-Aldrich, Merck KGaA, Darmstadt, Germany) and distilled water and stored at −80°C. Among six treated colonies, three received syrup contaminated with 50 ppb Imidacloprid (Sigma-Aldrich, Merck KGaA, Darmstadt, Germany, PESTANAL, CAS 138261-41-3) with 50 ppm acetone and three received syrup contaminated with 4.500 ppb Thiacloprid (Sigma-Aldrich, Merck KGaA, Darmstadt, Germany, PESTANAL, CAS 111988-49-9) with 50 ppm acetone. Three control colonies were fed sugar syrup containing 50 ppm of acetone.

### Electroantennography

The EAG recordings were conducted in two periods, in autumn 2018 (September 11–October 6) and spring 2019 (March 19–April 6). Forager bees carrying pollen loads were individually collected at the hive entrance with 60 ml transparent polypropylene vials (Nalgene™, Thermo Scientific, USA). The vials were previously rinsed with ethanol (90%) to remove any odor from the previous batch of bees. The vial opening was closed with a fine plastic mesh to allow full air circulation. The bees were provided sucrose sugar water (50% w/v) *ad libitum* through a 1.5 ml Eppendorf vial (Sigma-Aldrich, Merck KGaA, Darmstadt, Germany) with removed bottom, inserted into a hole in the mesh. The sugar water was made anew and refilled every morning. The vials with the bees were kept in the laboratory at a stable temperature of 20 ± 1°C at least 1 day before the EAG recording. Before each recording, the selected bee was anesthetized by leaving the vial in a fridge (4°C) for circa 60 s until immobilization. The head of the bee was removed with a scalpel, and the right antenna excised at the base of the scape. To avoid effects related to olfactory lateralization, the right antenna was used for every recording (22). The antenna was further clipped after the pedicel and the distal tip of the flagellum was removed. The base of the antenna was then mounted in a glass capillary filled with Ringer solution with 1% Polyvinylpyrrolidone (PVP average MW 360000 Da, Sigma-Aldrich, Merck KGaA, Darmstadt, Germany) and inserted in the indifferent electrode on the micromanipulators of an electrophysiological setup (Syntech GmbH, Buchenbach, Germany). The antenna tip was connected to the recording electrode. A batch of Ringer solution (7.5 g NaCl, 0.35 g KCl, 0.28 g CaCl_2_·H_2_0 dissolved in 1 l demineralized water) was previously prepared and aliquoted in single-use Eppendorf vials and stored at −80°C. A new vial of the solution was used every day. The manipulator base was connected to ground to minimize electrical interference. A polymethyl methacrylate (PMMA) box was used to cover the EAG setup and exclude airflows. A stable baseline on the oscillograph of the software indicated a successful contact between the antenna and the electrodes.

#### Coupled Gas Chromatography-Electroantennographic Detection Continuous Recording

The electroantennography detector described above was coupled with an Agilent 7820A chromatograph (GC) equipped with a Flame Ionization Detector (FID). Two μl of the pooled apple flower volatiles were injected into the GC capillary column HP-5 Agilent 19091J-413 of 0.25 μm coating, 30 m length, and 0.32 mm diameter. Helium at a flow rate of 2.5 ml/min was used as carrier gas. The oven method was programmed as follows: inject at 50°C and hold for 1.8 min, heating at 7.3°C/min until 250°C and hold for 3 min. The temperature of the injector was 250°C and the detector temperature was set to 350°C. The column effluent was mixed with a helium make-up (15 ml/min) and split at a 1:1 ratio, one part flowing to the FID, the other going through a transfer line (170°C) (Syntech GmbH, Buchenbach, Germany) into a charcoal-filtered and humidified airstream which was channeled to the mounted antenna. Before and after each recording, the mounted antenna was tested with a positive control (citral 10^−2^) according to the technique reported below, in order to assess the responsiveness of the antennal preparation. 50 μl of apple flower VOCs were taken from each of the CLSA elution vials and pooled together to avoid specimen differences and to create a working sample.

#### Discontinuous Recording (EAG)

**Figure 1:**
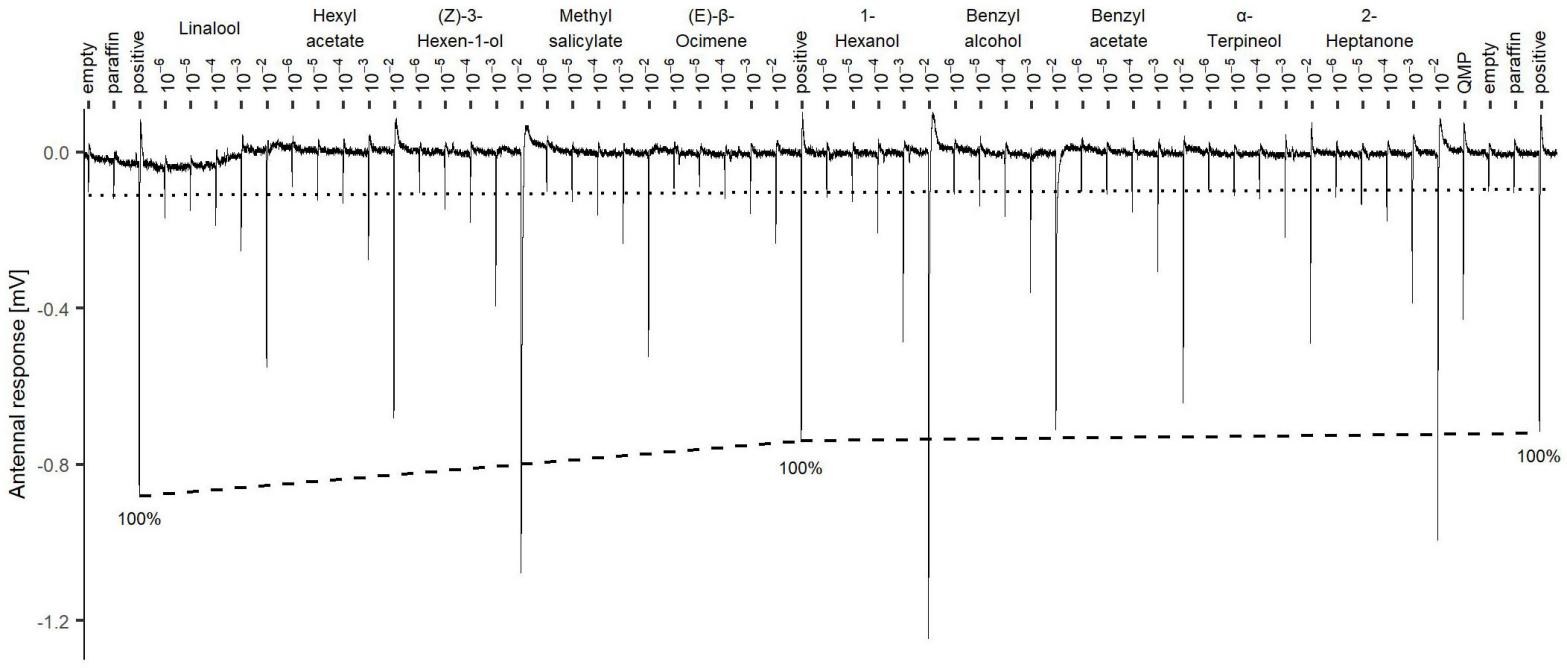
Stimuli presentation sequence for each antennal recording (example from Subject 4). Tested compounds are presented from lowest (10^−6^) to highest concentration 10^−2^ at 40 seconds intervals. Dashed line connects the responses to the positive control citral 10^−2^ and shows the 100% reference for the responses normalization. The dotted line is the average value of the negative controls empty pipette and empty pipette with paraffin oil (paraf) at the beginning and at the end of the recording.

The testing compounds were delivered into charcoal-filtered and humidified air constantly flowing through a metallic tube (0.6 l/min) using a stimulus controller (Syntech GmbH, Buchenbach, Germany). Each of the compound standards (Sigma-Aldrich, Merck KGaA, Darmstadt, Germany) was dissolved in paraffin oil (Sigma-Aldrich, Merck KGaA, Darmstadt, Germany) at decreasing concentrations of 10^−2^, 10^−3^, 10^−4^, 10^−5^, and 10^−6^ (w/w). The solutions were stored at −80°C and used also for the spring experiment. 50 μl of each dilution were applied on a piece of filter paper (Whatman 1, Sigma-Aldrich, Merck KGaA, Darmstadt, Germany), cut to a size of 3 × 1 cm and W-folded, and inserted into a glass Pasteur pipette (Sigma-Aldrich, Merck KGaA, Darmstadt, Germany). Immediately after the application, the pipette openings were sealed with Parafilm (Sigma-Aldrich, Merck KGaA, Darmstadt, Germany) to prevent dispersion of the volatile compound. The prepared pipettes were singularly marked and stored at −80°C, to use a new batch for each antennal recording. The pipette batch was taken out of the freezer 1 h before the recording, to reach room temperature. The interval between stimuli was 40 s, and the stimulus duration was 0.5 s. The pipette was inserted into the metal tube through a hole at a distance of 10 cm from the antennal preparation to ensure the mixing of the stimulus with the airflow. During the stimulus, the continuous carrier airflow was reduced by the same amount that the stimulus added, thus keeping a constant stream reaching the antenna. Each antenna was presented with two negative controls, one of pure air (empty pipette) and one of filter paper and paraffin oil only, at the beginning and the end of the trial. A positive control (citral at 10^−2^) was presented after the negative controls at the beginning, the end, and in the middle of the recording. If the response of the mounted antenna was < −0.5 mV at the beginning or < −0.2 mV at the end, the preparation was discarded and another antenna was prepared. The testing VOCs were presented sequentially from the lowest (10^−6^) to the highest (10^−2^) concentration. The order of presentation was: linalool, hexyl-acetate, *(Z)*-3-hexen-1-ol, methyl-salicylate, *(E)*-β-ocimene, 1-hexanol, benzyl-alcohol, benzyl-acetate, α-terpineol, 2-heptanone (Figure 1). The same order was used in both Autumn and Spring recordings. The antennae were also presented with a substitute of the queen mandibular pheromone complex (QMP) (Bee Boost, Savorelli, Italy), by inserting the plastic tube that contained it directly into a glass Pasteur pipette. Each recording lasted approximately 40 min in total. Twenty-four bees (8 per hive) were tested per treatment in the autumn, and 12 bees (4 per hive) in the spring. Since bees were collected from 9 different hives (3 hives × 3 treatments), the bee antennae were tested following a fixed order, thus controlling for possible time-related effects due to the date of collection or date of recording.

### Identification of the Bioactive Flower Volatiles

Two μl of the pooled apple flower volatiles were injected in a splitless mode in a GC system (7890A) coupled with an MS (5975C Network) (Agilent Technologies, Santa Clara, USA) for identification of the compounds. The GC column was a non-polar HP-5MS (Agilent Technologies), 0.25 μm coating, 30 m length, and 0.25 mm diameter. Helium at a flow rate of 1.2 ml/min was used as carrier gas. The oven method was programmed as follow: inject at 50°C and hold for 1.5 min, then 7.5°C/min until 250°C and hold for 5 min. The temperature of the injector was 250°. The GC-MS data acquisition and analysis were performed using the ChemStation software (Agilent Technologies). Compounds were initially identified by mass spectra comparison with the databases NIST 14 (Gaithersburg, MD, USA) and Wiley 7 N (Wiley, Hoboken, NJ, USA). Linear retention indices (LRI) of the peaks (23) were calculated by using a mixture of n-alkane standards (nC9-nC20, Sigma-Aldrich, Milan, Italy). The obtained LRI values were compared with reference LRI values available in the literature. Since the GC-MS and the GC-FID used in the experiments had different column diameters, the methods were designed to match the alkanes retention times at the best possible. Furthermore, the correspondence between the bee antennal responses and the GC peaks was confirmed by the respective LRI. Finally, the identity of compounds was confirmed by comparing the mass spectra and the retention times with those of authentic standard compounds (Sigma-Aldrich, Merck KGaA, Darmstadt, Germany).

### Data Analysis

#### Normalization of Response Values

As a positive control, citral was presented at the beginning, middle, and end of each recording using the concentration 10^−2^. Following standard procedure [e.g., (24)], the data were normalized by setting the response to the positive controls to 100% and calculating a linear trend for each bee antenna (Figure 1). Subsequently, the observed stimulus values were normalized according to the positive control trend line and expressed as percentages.

#### Statistical Analyses

The statistical analyses were performed using the software R (25). The packages emmeans (26), MASS (27), lmerTest (28) and lme4 (29) were used for analyses, while ggplot2 (30) was used for graphics.

The statistical analysis considered three effects:

- Short-term effects of treatments: To test for the effect of neonicotinoid treatment in the weeks after administration, only the autumn recordings were employed, by comparing each treatment group to the control group.
- Long-term effects of treatments: To test for the effect of neonicotinoid treatment several months after administration, the spring recordings were employed, again comparing each treatment group to the control group.
- Season effect: Variations in the antennal responses between autumn and spring were tested by comparing only antennal recordings obtained with the control group.

For each of these effects (short-term, long-term, season), the following analyses were performed:

i. The decay in antennal responses to negative and positive controls was analyzed using linear mixed-effects models (LMM) with the difference in response between the measurements at the beginning and end of each recording as dependent variable, season or treatment as fixed effects, and hive ID as random effect. For each fixed effect, three models were run analyzing the antennal responses to positive controls, to negative controls using empty pipettes, and to negative controls using paraffin oil, respectively.
ii. Antennal responses to the substitute of the queen mandibular pheromone complex were analyzed using an LMM with season or treatment as fixed effects, and hive ID as random effect.
iii. The antennal responses to VOCs were analyzed separately for each concentration of a VOC using LMMs with season or treatment as fixed effects, and hive ID as random effect.

Model diagnostics of LMMs were produced and evaluated using the DHARMa package (31). The random effect associated with bee hive ID explained on average 3% of the variance in the seasonal models (minimum = 0%, maximum = 70%), 11.9% of the variance in the short-term effect models (minimum = 0%, maximum = 38.4%), and 2.5% of the variance in the long-term effect models (minimum = 0%, maximum = 46.2%). Random effects were removed if they explained zero variance (*i.e*., converting LMMs into linear regression models), otherwise, they were retained in the models. *P*-values were computed from LMMs using Kenward-Roger estimation of the number of degrees of freedom. *P*-values were corrected for multiple tests within each type of VOC (*i.e*., tests on observations at different VOC concentrations) using the “false discovery rate” method (32).

To test whether the response to VOCs differed from the response to negative controls, for each concentration of a VOC, the normalized responses among all subject bees were compared to the normalized responses to the negative controls using paired *t*-tests.

Response values in the text and figures are reported as means of normalized antennal responses ± one standard deviation unless otherwise specified. The term “response signal” is used to indicate non-normalized values.

## Results

### Headspace Volatile of Apple Flowers

We used chromatographic analysis to identify a set of 18 flower VOCs typical of the flower samples (Table 1). These compounds were reported in other publications as commonly emitted from apple flowers. p-Xylene and 3-heptanone were reported in Baraldi et al. (33) as released by the flowers of different fruit tree species. Benzaldehyde, hexyl acetate, limonene, linalool, benzyl acetate and decanal were identified as main compounds of apple flowers (34). (*E*)-2 hexen-ol, phenol, 1-phenylethanone, benzyl alcohol, 1-ethenyl-3-ethyl-benzene, cinnamaldehyde, 3-methyl-1-ethynylbenzene, Z-3-hexenyl benzoate were found with either CLSA or SPME technique in apple flowers (35). Heptadecane and anisole were reported specifically from Fuji apples (36, 37).

**Table 1:** Volatile compounds identified by GC/MS analysis of the headspace of *Malus domestica* cv Fuji flowers and their amounts (10^−4^ TIC).

**Compound**	**LRI^**a**^**	**LRI^**b**^**	**10^**−4**^ TIC**
(*E*)-2-Hexen-ol	852	850^1^	354.2
p-Xylene	865	865^†^	81.6
3-Heptanone	869	869^1^	88.9
Anisole	916	916^1^	156.8
Phenol	948	948^1^	17.3
Benzaldehyde	962	963^†^	33.8
Hexyl acetate	1012	1012^1^	117.1
1-Phenylethanone	1033	1032^1^	61.8
Limonene	1039	1034^†^	1637.3
Benzyl alcohol	1044	1039^†^	332.7
1-Ethenyl-3-ethyl-benzene	1063	1064^1^	49.1
Linalool	1104	1101^†^	232.3
Benzyl acetate	1170	1167^†^	203.2
Decanal	1214	1207^†^	28.0
Cinnamaldehyde	1312	1305^1^	47.6
3-Methyl-1- ethynylbenzene	1453	1451^2^	31.5
Z-3-Hexenyl benzoate	1573	1571^1^	29.3
Hexadecane	1603	1600^†^	38.3

*LRI, Linear retention index calculated in relation to n-alkanes*.

*LRIb, Linear retention index already published in peer-reviewed journals and listed in PubChem (1) or Pherobase (2). When possible, the LRI was verified by a standard compound (^†^)*.

*The volatile compounds were collected in April 2018 by CLSA technique for 3 h. The compounds were identified by mass spectrometry and confirmed by linear retention indexes from the literature or laboratory standards when available*.

### EAD Responses to Flower Volatiles

**Figure 2:**
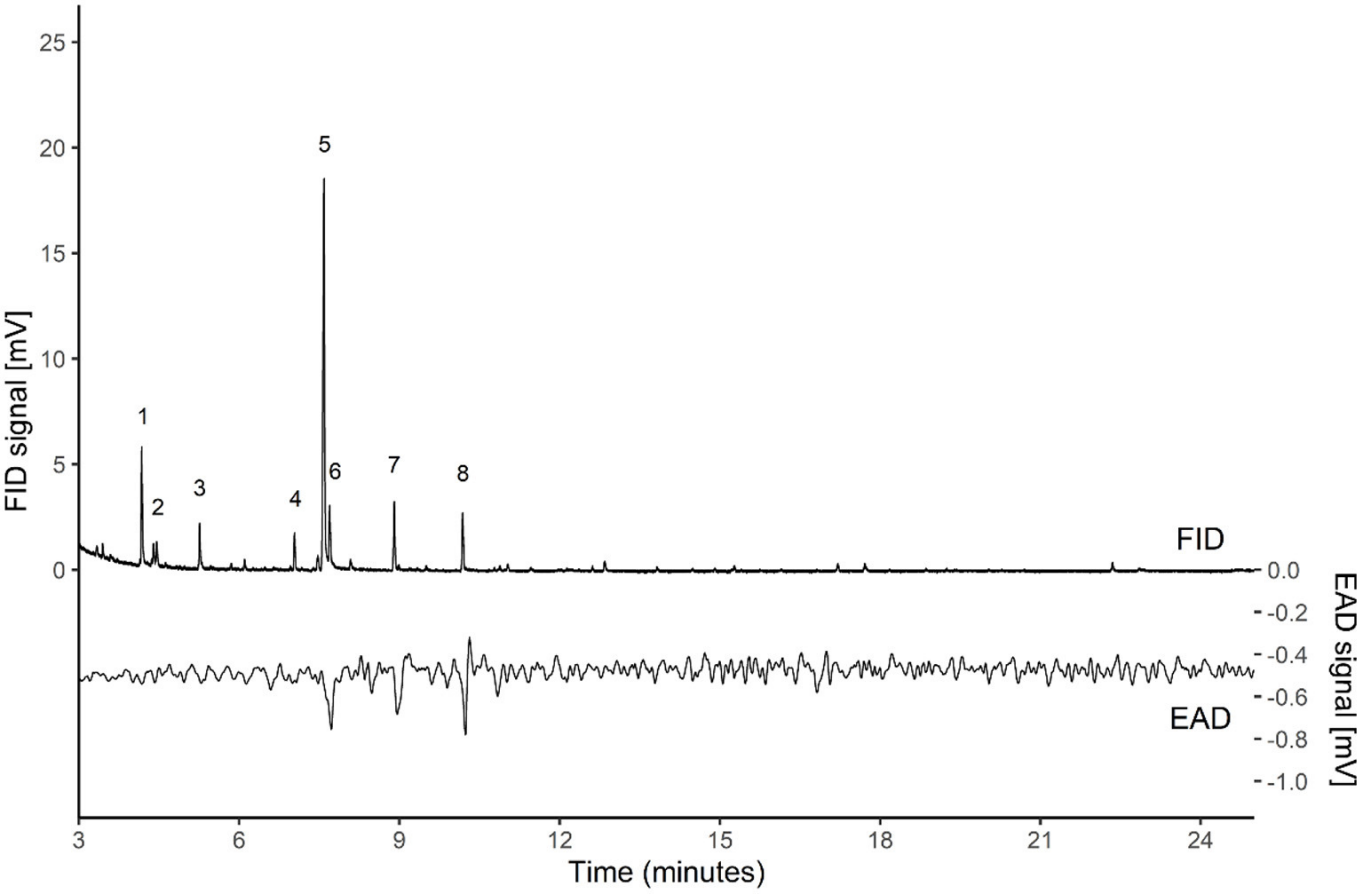
GC-EAD recording of a bee antenna (lower line) responding to the headspace volatiles (upper line) of apple flowers cv Fuji, collected in May 2018 with CLSA technique for 3 h. In multiple (6 out of 13) recordings, clear signal deflections were observed in correspondence to the VOCs reported in the figure: 6. benzyl alcohol, 7. linalool and 8. benzyl acetate. The other main peaks were: 1. (*E*)-2-hexenol, 2. p-xylene, 3. anisole, 4. hexyl acetate, 5. Limonene.

Three apple flower VOCs elicited clear antennal responses: benzyl alcohol, linalool, and benzyl acetate (Figure 2). The responses were confirmed in 6 out of 13 antennal recordings.

These three VOCs were employed in the EAG discontinuous recordings, and six other common flower VOCs were also added to the experiment: 1-hexanol, α-terpineol, methyl-salicylate, hexyl-acetate, *(E)*-β-ocimene, and *(Z)*-3-hexen-1-ol. Moreover, also the alarm pheromone 2-heptanone and a substitute of the queen mandibular pheromone were included in the trials.

### EAG Responses to Flower Volatiles

#### Compound Dependence

**Figure 3:**
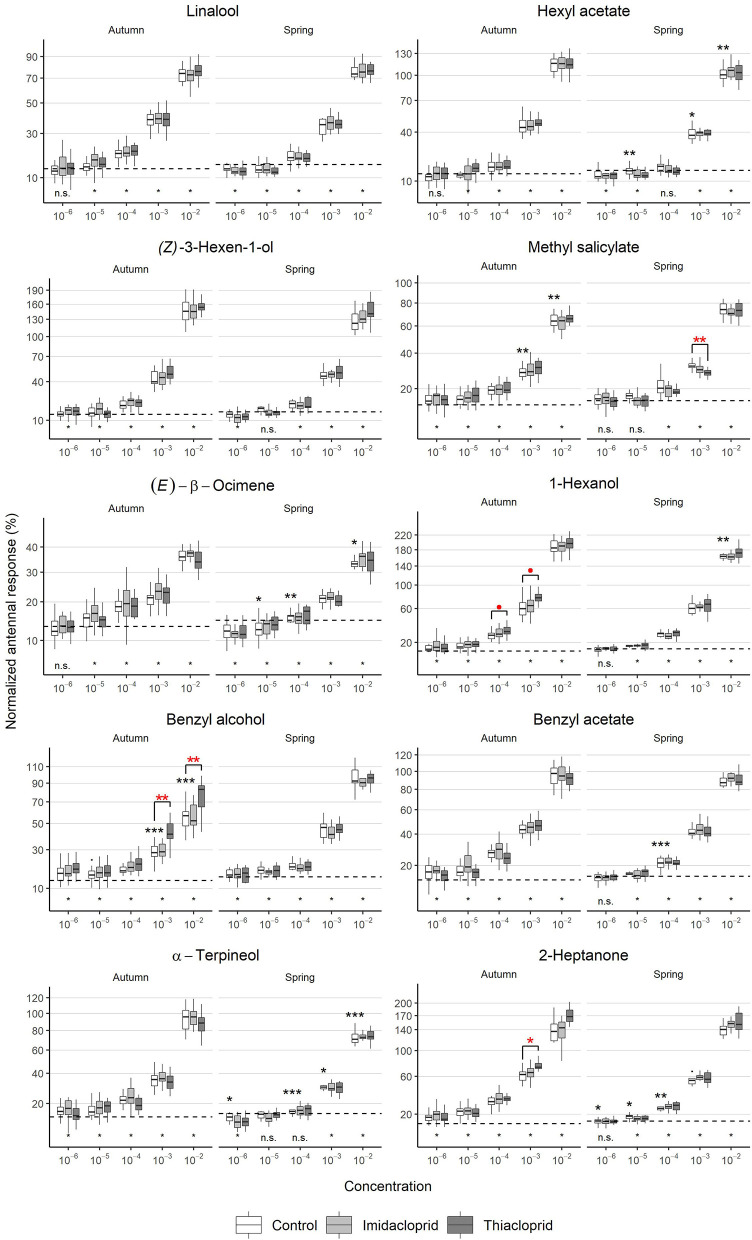
Antennal dose-response curves to flower volatiles during different seasons. Autumn recordings started 2 weeks after the end of the treatments and reveal the short-term effect of the treatments. Spring values, recorded 7 months after the exposure, show the long-term effects of the treatments. Normalized EAG signals as a function of the season [bees collected in September (*N* = 72) or April (*N* = 36)], Treatment (Imidacloprid 50 ppb or Thiacloprid 4500 ppb in sugar syrup, Control: pure syrup). Black asterisks report statistical significance between seasons (controls in autumn vs controls in spring), whilst red asterisks report statistical significance between treatments and controls within the same season (LMM). *p* ≈ 0.05, *p < 0.05, **p < 0.01, ***p < 0.001). The dashed lines show the average response to the negative controls (empty pipette and solvent) presented at the beginning of the recording. The asterisks below it indicate whether the responses to the concentration of each compound are significantly different than the responses to the negative controls (LMM, * = significant, n.s. = not significant).

The EAG experiments showed a dose-dependent antennal response (Figure 3). The VOC stimuli were presented from low to high concentration and normalized response values increased at a rate of ~3-fold with each 10-fold increase in concentration, except for the lowest concentrations 10^−6^ and 10^−5^ for which similar responses were observed. Furthermore, the strength of the antennal response depended on the VOC. The strongest antennal responses were elicited by the compound 1-hexanol, with a normalized response of 184 ± 24% at the concentration 10^−2^; twice the response to the positive control citral. *(E)*-β-ocimene elicited the weakest antennal responses, with a normalized response of 35.6 ± 4.8% at the concentration 10^−2^; approximately one third of the response to the positive control. The remaining flower VOCs elicited intermediate responses.

#### Season Effect

The EAG normalized responses to VOCs in the control group varied between seasons in different ways (Figure 3). For some of the VOCs, responses were weaker in spring than in autumn at multiple concentrations:

- Hexyl acetate elicited weaker responses in spring at concentrations 10^−3^ (LMM: *t* = −2.65, *d.f*. = 30.08*, p* = 0.021) and 10^−2^ (linear regression model: *t* = −3.57, *d.f*. = 33*, p* = 0.0055).
- *(E)*-β-ocimene elicited weaker responses in spring at concentrations 10^−5^ (linear regression model: *t* = −2.84, *d.f*. = 33, *p* = 0.015), 10^−4^ (LMM: *t* = −3.40, *d.f*. = 30.95, *p* = 0.0093) and 10^−2^ (linear regression model: *t* = −2.79, *d.f*. = 33, *p* = 0.015).
- 1-Hexanol elicited a weaker response in spring at concentration 10^−2^ (LMM: *t* = −3.60, *d.f*. = 31.09*, p* = 0.0054).
- Benzyl acetate elicited a weaker response in spring at concentration 10^−4^ (linear regression model: *t* = −4.30, *d.f*. = 33*, p* < 0.001).
- α-terpineol elicited weaker responses in spring at concentrations 10^−6^ (linear regression model: *t* = −2.25, *d.f*. = 33*, p* = 0.039), 10^−4^ (linear regression model: *t* = −4.73, *d.f*. = 33*, p* <0.001), 10^−3^ (linear regression model: *t* = −2.32, *d.f*. = 34*, p* = 0.039), and 10^−2^ (linear regression model: *t* = −4.65, *d.f*. = 34*, p* < 0.001).

In six cases, increased responses were observed in spring:

- Hexyl acetate elicited a stronger response in spring at concentration 10^−5^ (LMM: *t* = 3.32, *d.f*. = 27.12, *p* = 0.0064).
- Methyl salicylate elicited stronger responses in spring at concentrations 10^−3^ (linear regression model: *t* = 3.96, *d.f*. = 33*, p* = 0.0018) and 10^−2^ (LMM: *t* = 3.74, *d.f*. = 32.00*, p* = 0.0018).
- Benzyl alcohol elicited stronger responses in spring at concentrations 10^−3^ (linear regression model: *t* = 6.93, *d.f*. = 33*, p* < 0.001) and 10^−2^ (LMM: *t* = 9.09, *d.f*. = 32.00*, p* < 0.001), with a trend toward significance at 10^−5^ (linear regression model: *t* = 2.08, *d.f*. = 32, *p* = 0.076).

#### Short-Term and Long-Term Treatment Effects

Effects of neonicotinoid treatment on antennal responses were only observed in the group exposed to Thiacloprid (Figure 3, Supplementary Table 1). Short-term effects were observed for two VOCs:

- Benzyl alcohol elicited increased responses in the group exposed to Thiacloprid compared to the control group at concentrations 10^−3^ (LMM: *t* = 4.28, *d.f*. = 6.00, p = 0.013) and 10^−2^ (LMM: *t* = 4.29, *d.f*. = 5.98, *p* = 0.013).
- For 1-hexanol, increased responses in the group exposed to Thiacloprid at concentrations 10^−4^ (linear regression model: *t* = 2.36, *d.f*. = 65, *p* = 0.052) and 10^−3^ (LMM: *t* = 3.52, *d.f*. = 5.96, *p* = 0.052) were close to significance.

In addition, one long-term treatment effect was detected, as Methyl salicylate elicited a lower response in the group exposed to Thiacloprid compared to the control group at concentration 10^−3^ (linear regression model: *t* = −3.55, *d.f*. = 33, *p* = 0.0058).

### Responses to Positive and Negative Controls

**Figure 4:**
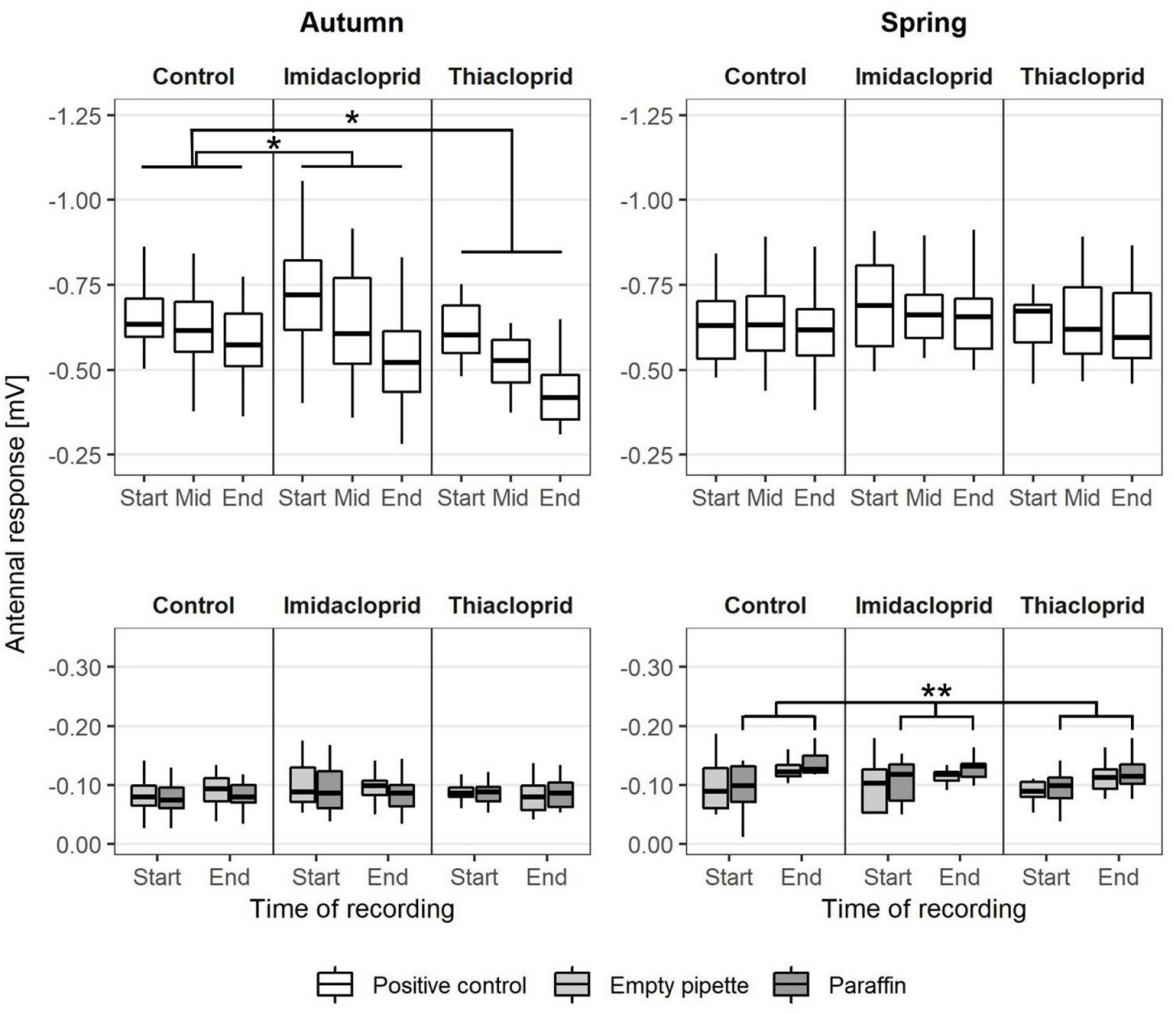
Temporal variation of the antennal response during the EAG recordings to the positive (citral 10^−2^) and the negative (empty pipette and pipette with paraffin) control stimuli. Values reported as a function of the season [bees analyzed in September (*N* = 72) or April (*N* = 36)], Treatment (Imidacloprid 50 ppb or Thiacloprid 4500 ppb in sugar syrup, Control: pure syrup) and the recording time within and experimental trial (start, middle, end) of a recording session. Asterisks report statistical significance (LMM, *p < 0.05, **p < 0.01).

The compound citral was presented as a positive control in a concentration of 10^−2^ at the start, the middle, and the end of each recording. Each recording lasted 39 min and the response signal to the positive controls showed that antennal responsivity decreased over time (Figure 4). The decays in antennal response signal from the beginning to the middle of each recording did not differ from the decays from the middle to the end of each recording, indicating that antennal response signals decreased linearly (paired *t-*test, *t* = −0.84, *df* = 107, *p* = 0.40). We observed no difference between autumn and spring in the response values to the positive controls in the control group. The responses to the first presentation of citral did not differ between treatments neither in the short-term nor in the long-term. However, we observed a short-term effect of neonicotinoid treatment in the decay of antennal response signal to the positive controls, as in the autumn it was significantly stronger in both the Thiacloprid- (LMM: *t* = 3.18, *d.f*. = 6.00, *p* = 0.019) and Imidacloprid-treated (LMM: *t* = 3.26, *d.f*. = 6.00, *p* = 0.017) groups compared to the control group (Supplementary Table 2). No long-term effect of treatment was observed, as the decay in response signal to the positive controls in the spring was similar across all groups.

The antennae also responded to the negative controls (empty pipette and pipette with paraffin oil on paper) (Supplementary Table 2). The negative controls were presented either at the beginning or the end of each recording, thus indicating a signal baseline (Figure 3). In the control group, the response signal to the negative controls did not differ between the beginning and the end of each recording in the autumn experiment. In the spring experiment, response values to the negative controls containing paraffin oil increased significantly during the time of recording (Figure 4) (linear regression model: *t* = 3.10, *d.f*. = 34, *p* = 0.0039), but such a pattern was not observed for the empty pipette (linear regression model: *t* = 1.28, *d.f*. = 34, *p* = 0.21). No short-term or long-term effects of neonicotinoid treatment were observed on the response signal to negative controls.

### Responses to the Pheromones

**Figure 5:**
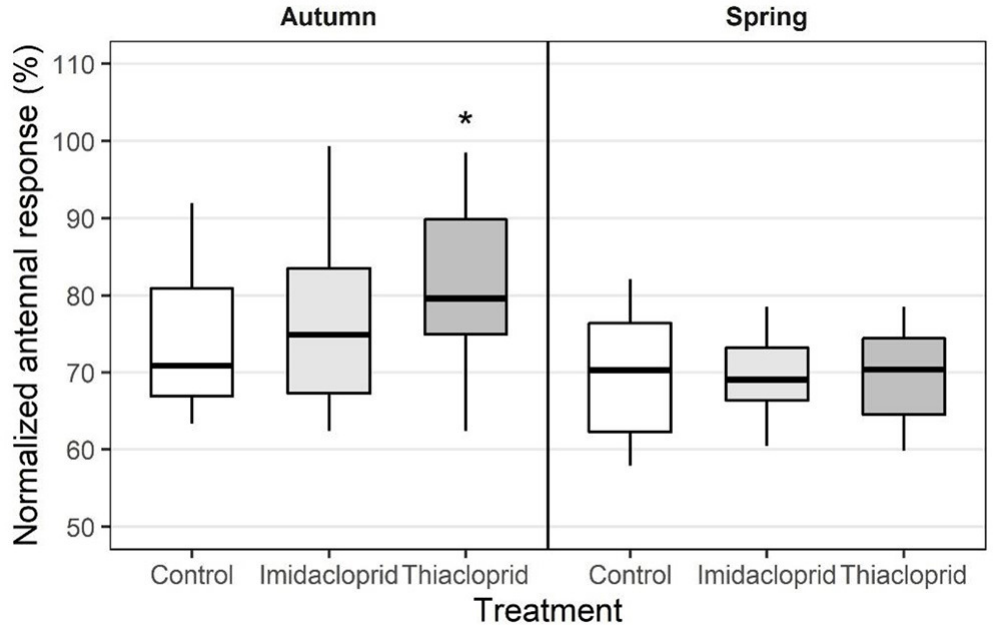
Antennal responses to Bee boost (Savorelli, Italy), a commercial substitute of the queen mandibular pheromone complex. Normalized EAG signals as a function of the season [bees collected in September (*N* = 72) or April (*N* = 36)], Treatment (Imidacloprid 50 ppb or Thiacloprid 4500 ppb in sugar syrup, Control: pure syrup). Asterisk reports statistical significance (LM, *p < 0.05).

When comparing the control group in autumn and spring, the normalized EAG response values to the QMP substitute did not change significantly with season. A short-term treatment effect was found in the group exposed to Thiacloprid, for which responses to the QMP substitute were significantly increased compared to the control group in the autumn (linear regression model: *t* = 2.11, *d.f*. = 69, *p* = 0.039) (Figure 5). No effect of treatment was observed in the long term, as responses to the QMP substitute in the spring were similar across all groups.

For 2-heptanone, the other pheromone tested in the trial (Figure 3, Supplementary Table 1), responses in the control group were weaker in spring for concentrations 10^−6^ (LMM: *t* = −2.66, *d.f*. = 29.98*, p* = 0.021), 10^−5^ (linear regression model: *t* = −2.82, *d.f*. = 34*, p* = 0.020), and 10^−4^ (linear regression model: *t* = −3.50, *d.f*. = 34*, p* = 0.0066), and with a trend toward significance for 10^−3^ (LMM: *t* = −1.95, *d.f*. = 32.00, *p* = 0.075). In addition, a short-term treatment effect was observed in the group exposed to Thiacloprid with a significant increase in responses to 2-heptanone at concentration 10^−3^ in the autumn (LMM: *t* = 3.84, *d.f*. = 5.91, *p* = 0.046). As evident from Figure 3, an increased response was also observed at concentration 10^−2^, however this effect was not significant after correction for multiple testing (LMM: *t* = 2.49, *d.f*. = 5.95, *p* = 0.12). No long-term effect of treatment was observed, as reponses to 2-heptanone in the spring were similar across all groups.

### Difference From the Negative Controls

At the lowest concentrations, the VOC dilution should reach a level of undetectability for which the antennal responses should approach a value around the average of the negative controls. However, in the spring trials, we recorded responses that were significantly lower than the responses to the negative controls for five VOCs (Figure 3):

- Linalool at concentrations 10^−6^ (paired *t*-test: *t* = −3.38, *d.f*. = 35, *p* = 0.0017) and 10^−5^ (paired t-test: *t* = −3.41, *d.f*. = 35, *p* = 0.0016).
- Hexyl acetate at concentrations 10^−6^ (paired *t*-test: *t* = −4.15, *d.f*. = 35*, p* < 0.001) and 10^−5^ (paired t-test: *t* = −2.75, *d.f*. = 35*, p* = 0.0091).
- *Z*-3-Hexenol at concentration 10^−6^ (paired *t*-test: *t* = −5.25, *d.f*. = 35*, p* < 0.001).
- *(E)*-β-ocimene at concentrations 10^−6^ (paired *t*-test: *t* = −4.81, *d.f*. = 35*, p* < 0.001) and 10^−5^ (paired *t*-test: *t* = −2.55, *d.f*. = 35*, p* = 0.015).
- α-terpineol at concentration 10^−6^ (paired *t*-test: *t* = −4.11, *d.f*. = 35*, p* < 0.001).

Responses to methyl salicylate, 1-hexanol, benzyl alcohol, benzyl acetate and 2-heptanone were never significantly lower than the negative control.

## Discussion

### Bioactive Compounds and Dose Sensitivity

The coupled gas chromatography-electroantennographic detection continuous recordings (GC-EAD) allowed us to identify three apple flower compounds, benzyl alcohol, linalool, and benzyl acetate, that elicited strong signal responses. These compounds are generally reported in apple flowers (34, 38, 39) and were shown to play a role as bioactive compounds to honey bee antennae (40). To extend our investigation to a wider range of candidate flower volatiles, six more odorants were included in the study. Most of these were previously described in stone fruit flowers (41) and apple flowers, such as *(Z)*-3-hexen-1-ol (34, 40), hexyl-acetate, *(E)*-β-ocimene, methyl salicylate (34), 1-hexanol (39). α-terpineol was found in other plants of honey bee interest like black locust (42), field scabious (43) and St. John's wort (44).

In our recordings, the strength of the antennal response depended on the compound and its concentration, *e.g.*, 1-hexanol and *(Z)*-3-hexen-1-ol elicited overall higher responses than other VOCs. Such variations are intuitive and widely reported in the literature for honey bees (45–47) and other insects (48, 49). It is worth noting that, as pointed out by Andersson et al. (50), the amount of a volatile that reaches the olfactory sensory neurons depends on the intrinsic nature of the molecule and its affinity with the solvent. Therefore, the observed responses might have been influenced by different airborne quantities. For this study, however, comparisons between volatiles were not relevant as we focused on the effect of the neonicotinoid treatments and season on the response to each individual volatile.

Five of the flower volatiles employed in the study have also been associated with pheromone functions in honey bees. Citral consists of the two isomers (*E*)-citral and (*Z*)-citral, which are components of a pheromonal blend with attractive effect released by the Nasonov gland (51). The volatiles 1-hexanol, hexyl acetate, benzyl alcohol, and benzyl acetate are found in the honey bee alarm pheromone emitted by the stinging apparatus (52). However, 1-hexanol and hexyl acetate (53) appear to be involved in nestmate recruitment rather than stinging behavior (54). Benzyl alcohol was reported as being ineffective as an alarm pheromone (55), e.g., it did not elicit defensive behaviors (56), and its function as a pheromone seems unclear (54). Benzyl acetate alone only induced flight behavior (57), and had a repellent effect when combined with n-octyl acetate (54, 58). Meanwhile, all of these compounds are extremely common flower volatiles, and they may be employed by plants to attract bees by mimicking their pheromones (54).

### Seasonal Effects

Bees face different organizational tasks and accordingly undergo physiological changes between autumn and spring (59). Whether these changes may involve the peripheral reception is, so far, unknown, and there is a gap in the literature regarding the variation of olfactory responses with season. One study examined the annual sensitivity dynamics of honey bee workers, focusing, however, only on the queen extract (60), but other pheromones or flower volatiles have not previously been investigated. In the present study, we report that seasonal variations in responses to VOCs and pheromones occurred at multiple concentrations. The variation between our autumn and spring recordings suggests a seasonal change in honey bee perception of VOCs with antennal responses in some cases being weaker in spring than in autumn. However, confirming such a general trend would require a repetition of the experiment over multiple years. In Skirkevièius and Skirkevièienë (60), antennal sensitivity varied during the year, with pheromone receptors being more sensitive in May-July and January-March and less sensitive in April and August-December. In the periods that we carried out our trials for the present study (September-October and March-April), Skirkevièius and Skirkevièienë observed that antennae were less responsive to the queen extract with no significant variation between autumn and spring. In agreement with this, our results show no seasonal changes in antennal responsivity to the QMP, however, we did observe variations for the alarm pheromone 2-heptanone and the flower volatiles.

The reasons behind these seasonal variations are unexplored and may reflect changes in the insects' physiology as well as adaptations to environmental conditions. For instance, insect antennae are sensitive to humidity (61) and hygroreceptors are found in the mid-region of the honey bee antennal segments (62, 63). These receptors respond to changes in humidity and air pressure following the deformation of the dendritic membranes of the moist and the dry cells forming the receptor (64). They respond with oscillations in impulse frequency depending on the humidity and the rate of humidity change. During the recordings in the present study, antennae were exposed to a controlled airflow purified through a carbon filter and subsequently humidified in a water bubbler, thus ensuring a standardized treatment. However, Tichy and Kallina (63) showed that honey bee hygroreceptors are not very efficient in detecting instantaneous humidity changes, while they are rather specialized for large-amplitude fluctuations. Therefore, we cannot rule out the possibility that differences in the environmental relative humidity outdoors or in the laboratory, rather than in the air stream, might have affected the overall antennal sensitivity. Yet, since not all the responses were lower in spring (*i.e*., sensitivity to methyl-salicylate at 10^−2^ and 10^−3^ and benzyl-alcohol at 10^−2^ and 10^−3^ was higher in spring than in autumn), environmental conditions such as humidity would only contribute significantly to the seasonal effect if they affected some chemical receptors more than others, or even in opposite directions in terms of odor sensitivity. We consider that to be rather unlikely. Thus, it appears that the seasonal changes in antennal sensitivity are likely to be associated with physiological changes and not reflecting changes in environmental conditions.

We propose that such seasonal changes may be associated with a change in the number of sensilla and receptors according to the season (*i.e.*, winter or summer bee). Awad et al. (65) described the variations of sensilla present on the flagellum of young (3-days old) worker bees in different queen statuses (1-year-old mated queen; supersedure queen; 3-day-old virgin queen; queen cell; without queen; and without queen/without bee bread). They showed that the antennae exhibited significant changes in the number of the sensilla Chaetica, Placodea, and Trichodea between the different queen conditions. The number of sensilla may also change according to the bee type, as Riveros and Gronenberg (66) have shown that the number of olfactory sensilla is greater in pollen and water foragers, which are known to exhibit higher sensory sensitivity, compared to nectar foragers. Similarly, seasonal plasticity between generations of winter and summer bees is possible. Changes in the number of sensilla could be an ecological adaptation. As the availability of flowers strongly reduces in autumn, the low concentration of floral scents in the air might require bees to elevate their antennal sensitivity, while in spring, when flowers and their volatiles are abundant, less sensitivity is needed but the dynamic range needs to be higher. An investigation of the type, distribution, and number of sensilla during the year could reveal the drivers behind seasonal variation in antennal sensitivity.

### Short-Term vs. Long-Term Effects of Neonicotinoid Treatments

We detected neonicotinoid-induced changes in antennal responses to VOCs with effects varying between short- and long-terms. Treatment with Thiacloprid elicited an increase in antennal responses to benzyl alcohol and 1-hexanol as well as to the alarm pheromone 2-heptanone in the autumn experiment, while similar effects were not observed in the spring. In addition, treatment with Thiacloprid elicited decreased responses to methyl salicylate in the spring experiment, while a similar effect was not observed in the autumn.

The bees employed in the autumn experiment fed directly on contaminated treatment syrups, which were administered chronically at the end of the summer. In contrast, the bees employed in the spring experiment did not feed directly on the treatment syrups, but passed the winter feeding on stores made from the syrups administered the previous summer. Because all honey bees collected for the recordings were foragers at the hive entrance, we can exclude the scenario that autumn bees would have survived the winter to the following spring. The nectar, as the syrup, must undergo a series of chemical transformations before being stored (67), and these digestive processes contribute to degrading xenobiotics in the hive, possibly reducing the residual insecticide content in the final honey product (9). Honey bees can relatively quickly detoxify cyano-substituted neonicotinoids such as Thiacloprid, while nitro-substituted neonicotinoids, like Imidacloprid, are metabolized more slowly (68). This suggests that spring bees probably had access to a less toxic nutrition than bees in the autumn. We therefore expected that treatment effects would be more prominent in the autumn bees compared to the spring bees. Accordingly, we detected four out of five changes in antennal responses to floral odors in the Thiacloprid group in the autumn experiment and only one in the spring experiment. However, due to a reduction in sample sizes in the spring experiment, we had more statistical power to detect short-term compared to long-term effects, and our results should therefore be interpreted with caution.

### Thiacloprid-Induced Effects

Only one study has previously investigated the effect of neonicotinoids on antennae responses, however only in the short term. In a recent paper, Straub et al. conducted EAG recordings on *Osmia bicornis* L. (Hymenoptera, Megachilidae) and *Bombus terrestris* L. (Hymenoptera, Apidae) (24). After topical application of 0.75 μg and 2.55 μg Clothianidin for *O. bicornis* and *B. terrestris*, respectively, antennae were presented with two flower volatiles, 2-phenylethanol and linalool at different concentrations. Clothianidin reduced antennal responses to 2-phenylethanol, but the responses to linalool were not altered. These results correspond with our findings that treatment with Thiacloprid elicited variations in the antennal response only to some VOCs and some VOC concentrations. Straub et al. suggested that the neonicotinoids may affect receptors of only some chemical classes of VOCs, and recommended testing on a broader spectrum of compounds, as we eventually did. However, among the six Thiacloprid-altered responses in our data, the stimulus VOCs represented a rather diverse assortment of chemical classes: two were alcohols (1-hexanol and benzyl alcohol), one was an ester (methyl salicylate), and one was a ketone (2-heptanone). Thus, our results do not support the hypothesis that neonicotinoids affect receptors of only some chemical classes, and we recommend more research on the topic, *e.g.*, including calcium imaging of ORN signals in the antennal lobe (69).

The majority of studies on behavioral consequences of neonicotinoid exposure have reported an impaired ability to associate floral scents with food quality (70–75). Honey bees use flower scents to evaluate nectar quality, and subtle differences in the proportion of volatiles allow them to discriminate between floral scents (10, 76–78). Different concentrations of flower volatiles were reported to impact the visitation frequency of honey bees to sunflowers (79) and rapeseed (80) varieties. However, functional imaging of the antennal lobe response maps showed that neonicotinoids remove the odor-specificity of the response pattern, thus potentially disrupting the ability of bees to identify the plants with the best rewards (18). Accordingly, Clothianidin-treatment was found to decrease the number of flowers visited per foraging flight and increase both the duration of flower visits and the searching time between flowers in *Osmia bicornis* L. and *Bombus terrestris* L. (24). Similar results were observed for honey bees (81–85), including a disruption of the flight direction and inefficient foraging activity. An important result of our study is that treatment with Thiacloprid elicited an increase in antennal responses to benzyl alcohol and 1-hexanol. We propose that an enhanced sensitivity to certain flower volatiles above others may affect the ability of bees to accurately perceive the proportions of volatiles in floral scents, which may explain previous findings of effects of neonicotinoid exposure on foraging behavior of bees.

Altered antennal responses may also be involved in other behavioral consequences of neonicotinoid exposure. In particular, since benzyl alcohol and 1-hexanol have also been associated with pheromone functions in honey bees, regulatory effects of these VOCs on social behaviors such as aggression may play a role in addition to their function as floral scents. Furthermore, one study reported a neonicotinoid-induced increase in olfactory learning and memory (72). This is consistent with our findings, as an enhanced learning effect can be explained by a partial increase of the strength of odor responses, as observed in our study. The question of learning-induced changes in the EAG signal is all but clear, and the few existing studies report ambiguous results; some find a learning-induced increase of responses (86, 87), while other studies find no variations (88) or report decreased signals (89). To further explore whether pesticides may impair learning at the periphery of the olfactory system, combined studies of behavior and EAG responses are needed.

### Imidacloprid Induced No Effects

No differences were observed in the antennal responses between the Imidacloprid-treated group and the control group, neither in the short- nor long-term assessment (*i.e*., the autumn and spring experiments). The chosen treatment concentration of 50 μg/kg (ppb) in our experiment is above the usual amount found in the environment and in hive products (90–93) and can potentially be detrimental to the health of bee colonies (94). Previously reported effects include reduced foraging activity following exposure to Imidacloprid at 48 ppb for 4 days (95), reduced overwintering colony survival, higher rates of queen failure, and broodless periods at concentrations ranging from 20 to 100 ppb for 12 weeks (94), and reduced colony strength at a concentration of 100 ppb for 6 weeks (96). However, these colony effects may not be related to potential effects of Imidacloprid on the bee peripherical olfactory system. Thus, the observations in our study are not in contradiction with the previously observed colony effects.

Studies suggesting an Imidacloprid-induced impairment of the olfactory system are extremely limited. At the behavioral level, it was found that the administration of Imidacloprid at sublethal doses (0.1, 0.4, 1.1 ng) to the parasitic wasp *Nasonia vitripennis* (Walker) (Hymenoptera, Pteromalidae) disrupted sexual communication and host finding behavior (97). *In vitro* recordings from mushroom body Kenyon cells, showed that Imidacloprid and Clothianidin blocked neuronal firing (98). Calcium imaging of the honey bee antennal lobe projection neurons during exposure of the brain to a 10 μM solution of Imidacloprid showed an elimination of the odor-specific glomerular response patterns (18). However, from these studies, it is not possible to make inferences about effects at the level of the antennae or the ORNs.

### Affected Response Properties

The observed effects of treatment with Thiacloprid included both reductions and increases of antennal responses. In addition, the decays in antennal responses to positive controls during each recording increased in both neonicotinoid-treated groups. The observation of this decay was made possible because citral was the only VOC presented more than once, and the same may have been noted with other odors. However, while the observed signal variation suggests an effect of neonicotinoid treatment on honey bee olfactory sensitivity, the EAG technique applied in this study does not allow us to identify the mechanism behind the variation. EAG signals represent the summed potential change of receptors (99). Thus, any treatment-induced variation of the EAG signal may be explained by changes in the olfactory reception mechanisms or the antennal physiology. In the present experiment, the neonicotinoids could have affected either the number of receptors that were activated when cues were presented or the activation strength by altering the firing rate of single neurons. However, also physical and chemical modifications of the biological tissues could result in an altered electrical resistance between the neuronal site and the recording electrode (100).

A previous study has shown that Imidacloprid at sublethal doses interacts with ASP2, a general Odor-binding protein (OBP) in *Apis cerana*, and strongly decreased the binding of ASP2 to a common flower volatile, β-ionone (101). In addition, recent studies have reported altered expression of genes associated with mitochondria and oxidative phosphorylation in honey bees exposed to environmental concentrations of Thiacloprid (102) and Spinosad (103), potentially affecting cellular energy production. While the flux of fresh hemolymph in the antenna might compensate for such a detrimental effect in a living insect, the antennal excision and interruption of the flow of hemolymph could enhance the effect. Our results did not demonstrate an effect in living individuals, but they suggest a possible cellular disturbance that is likely to also cause an *in-vivo* impairment.

No difference was noticed in the responses to negative controls between neonicotinoid treatments and the control group. As most of the measured antennal responses to negative controls are likely produced by mechanoreception, the observed effects of neonicotinoid treatment in our study most likely do not affect the coding of mechanical stimuli (104). The fact that the responses to negative controls are significantly above the responses to some VOCs at the lowest concentrations is likely due to minimal odor contaminations of the control pipettes. When odor stimulus concentrations are low, small contaminations can significantly alter response signals and we believe that such an effect increased antennal responses to the negative controls in our experiment. The pipettes used for presenting the solvent (paraffin) on filter paper or dry filter paper (*i.e*., the negative controls) were used twice, at the beginning and end of each recording. The analysis showed that the response amplitudes significantly increased between the initial and the final presentation during the spring measurements, which is likely due to odor contamination in the second presentation. Because of this potential contamination, we are conservative in our interpretation of the responses measured at the lowest VOC concentrations (10^−6^ and 10^−5^).

### Effects on Pheromone Responses

Two compounds triggering a social response in honey bees were included in the present study. 2-heptanone is an alarm pheromone released by the honey bee mandibular glands (105) which induces a defensive (106) and a repellent (107) effect. It has been shown to impair learning and memory (15) and it has an anesthetic effect used to deter and paralyze intruders (108). In contrast, the queen mandibular pheromone (QMP) is an attractive blend of five diverse substances (109) that elicit the retinue response of worker bees (110). The QMP, in combination with other compounds such as queen esters and alcohols, act as primer pheromones that affect the physiology and behavior of worker bees (111). Because of their crucial role in colony social behavior, we considered these two pheromones as important candidates to include in our experiment.

Effects of pesticide exposure on the antennal responses to alarm pheromones have not previously been investigated. Meanwhile, increased aggressiveness of honey bees after exposure to neonicotinoids have been reported (112, 113). For example, Bortolotti et al. (114) reported an increased aggressiveness in 23.8% of affected hives after intoxication with neonicotinoids during spring 2008. Increased aggressivity due to neonicotinoids has also been described in ants (Hymenoptera: Formicidae), affecting competition and spread of invasive species (115). Our study shows an increased antennal response to the alarm pheromone 2-heptanone at concentration 10^−3^ in honey bees in the short-term following treatment with Thiacloprid, and a non-significant effect at concentration 10^−2^. Among five flower VOCs that have also been associated with pheromone functions, two (1-hexanol and benzyl alcohol) elicited an increased response in Thiacloprid-treated bees in the short term. These two compounds are part of the complex alarm pheromone blend emitted by the sting gland. The increased sensitivity to 2-heptanone, 1-hexanol, and benzyl alcohol suggests a general increased sensitivity to alarm pheromones in honey bees following exposure to Thiacloprid, which could induce abnormal responses, potentially triggering aggressive behaviors reported in the literature. However, we propose this hypothesis with caution, as the antennal responses to other components of the alarm pheromone blend (hexyl acetate and benzyl acetate) were not affected by treatment with Thiacloprid in our experiment. In addition, the function of benzyl alcohol as an alarm pheromone is uncertain. Further EAG experiments focusing on the alarm pheromone blend may shed more light on the observed alteration of antennal responses.

Our study also revealed a seasonal variation in the responses to 2-heptanone, as the response signals of the control group were generally higher in autumn than in spring. We propose that the higher responses to 2-heptanone in the autumn may be associated with a higher level of vigilance, which may be adaptive in honey bees. In autumn, the colonies are still strong in size, but as food availability plummets, it is vital for bees to preserve food storages and defend the colony against robberies typical of this period (116, 117). In contrast, spring bees experience abundant food resources, reducing the likelihood of robbing and the need for aggressive behavior. Thus, increased sensitivity to low concentrations of 2-heptanone in autumn bees may reflect an ecological adaptation.

As also observed for 2-heptanone, higher responses to the QMP in the short term were elicited in bees exposed to treatment with Thiacloprid. This increased sensitivity could reflect a compensatory mechanism triggered by compromised queen bees. Exposure to neonicotinoids has previously been reported to be associated with impaired and reduced fecundity (118, 119), immunosuppression (120), and reduced survival (121) in queen bees. High supersedure rates (60%) in autumn were observed in colonies exposed for 46 days to Thiametoxam and Clothianidin in summer (16), and this effect was ascribed to a reduced performance of exposed queens. A reduced metabolic rate (~11%) reported in queens exposed to Imidacloprid (122) might imply a reduced secretion of QMP, as its production and release involve an energy cost. Worker bees might perceive the queen impairment, if resulting in a lower pheromonal production, and compensate for it by increasing the sensitivity to avoid risky autumn supersedure.

Among honey bees exposed to Thiacloprid or Imidacloprid in our experiment, antennal responses to citral decreased significantly with the order in which it was presented, compared to controls. However, as EAG recordings were conducted on excised antennae, this effect cannot be inferred to antennae on living bees. Notably, antennal responses to the first presentations of citral were not affected by treatment with Thiacloprid or Imidacloprid and did not show any seasonal variation. Citral is a component of the Nasonov blend and our results imply that neonicotinoid exposure does not affect the sensitivity of honey bees to this aggregation pheromone.

A last consideration on bee pheromones will be given to β-ocimene. Although it is a common plant volatile released by several plant parts, including leaves (123) and flowers [e.g., (34)], β-ocimene plays a key role as a brood pheromone inside the beehive (124). It is produced by young larvae and expresses the nutritional needs of the brood. It inhibits ovary development in workers and stimulates early foraging in nest workers, thus regulating the food availability to the brood. In our study, β-ocimene elicited significantly higher responses in autumn than in spring for the control group. Because of its double role, as a plant volatile and as a pheromone, it is difficult to disentangle the causes and behavioral outcomes of such a difference. Yet, most likely, because of the reduced brood size in autumn, bees might tune their sensitivity to perceive even small amounts of the pheromone, while, in spring, the much larger brood surface allows for a less accurate detection.

## Conclusion

The present study reports variations in the antennal responses to flower volatiles and pheromones in honey bees chronically exposed to two neonicotinoids. In the short term, the antennae of bees exposed to Thiacloprid showed increased responses to the flower volatiles benzyl alcohol and 1-hexanol, to the queen mandibular pheromone, and to the alarm pheromone 2-heptanone. In contrast, only one long-term effect was observed, as spring bees from colonies exposed to Thiacloprid during late summer showed decreased responses to methyl salicylate–however only at one concentration. Treatment with Imidacloprid induced no changes in antennal responses to any of the tested VOCs or pheromones, neither in the short nor the long term. However, the antennae of bees exposed to Imidacloprid and Thiacloprid showed an increased signal degeneration in the short term. In addition, we observed season-related differences in antennal responses for seven VOCs and the alarm pheromone 2-heptanone.

The difference in observed treatment effects between the autumn and spring experiments indicate that the adverse effects of exposure to neonicotinoids may be stronger in bees that are directly exposed (acute effects) and less severe in subsequent generations. This difference between short- and long-term effects may hold promises of a brighter future for bee populations if agricultural practices that reduce the use of pesticides are widely implemented. However, the mechanisms behind the observed effects are yet unknown and we hope that the results of our study will stimulate further investigations of neonicotinoid effects on peripheral olfactory responses and antennal transduction in bees. It would be of great importance to link changes in antennal signals to behavioral observations at the single bee and colony level.

## Data Availability Statement

The original contributions presented in the study are included in the article/Supplementary Material, further inquiries can be directed to the corresponding author.

## Author Contributions

RF, SA, and AH conceived the research. RF and SA managed the bee colonies. RF conducted the experiments and the data collection. RF and JR conducted the data analysis. RF, SA, AH, and JR wrote the manuscript. All the authors read and approved the manuscript.

## Funding

This study was funded by the project TN2214 STEFANIE financed by the Autonomous Province of Bolzano (Italy).

## Conflict of Interest

The authors declare that the research was conducted in the absence of any commercial or financial relationships that could be construed as a potential conflict of interest.

## Publisher's Note

All claims expressed in this article are solely those of the authors and do not necessarily represent those of their affiliated organizations, or those of the publisher, the editors and the reviewers. Any product that may be evaluated in this article, or claim that may be made by its manufacturer, is not guaranteed or endorsed by the publisher.

## References

[1] Aizen MA Aguiar S Biesmeijer JC Garibaldi LA Inouye DW Jung C. Global agricultural productivity is threatened by increasing pollinator dependence without a parallel increase in crop diversification. Glob Chang Biol. (2019) 25:3516–27. 10.1111/gcb.14736 31293015 PMC6852307

[2] Henríquez-Piskulich PA Schapheer C Vereecken NJ Villagra C. Agroecological strategies to safeguard insect pollinators in biodiversity hotspots: chile as a case study. Sustainability. (2021) 13:6728. 10.3390/su13126728

[3] Khalifa SAM Elshafiey EH Shetaia AA El-Wahed AAA Algethami AF Musharraf SG. Overview of bee pollination and its economic value for crop production. Insects. (2021) 12:688. 10.3390/insects12080688 34442255 PMC8396518

[4] Montanarella L Panagos P. The relevance of sustainable soil management within the European Green Deal. Land Use Policy. (2021) 100:104950. 10.1016/j.landusepol.2020.104950

[5] Durant JL Ponisio LC. A regional, honey bee-centered approach is needed to incentivize grower adoption of bee-friendly practices in the almond industry. Front Sustain Food Syst. (2021) 261:628802. 10.3389/fsufs.2021.628802

[6] Vanbergen AJ Baude M Biesmeijer JC Britton NF Brown MJF Bro M. Threats to an ecosystem service: pressures on pollinators. Front Ecol Environ. (2013) 11:251–9. 10.1016/S0021-9673(01)80947-X 14062605

[7] Favaro R Luke A Antonacci G Rizzi E Preti M Angeli S. Honeybees as pesticide monitoring tools? A multi-stakeholder community project in an Alpine valley offers promise for mapping pesticide dispersal. (2022). [Manuscript in preparation].

[8] Manzoor F Pervez M. Pesticide impact on honeybees declines and emerging food scurity crisis. In global decline of insects. IntechOpen. (2021). 10.5772/intechopen.98871

[9] Xiao J He Q Liu Q Wang Z Yin F Chai Y. Analysis of honey bee exposure to multiple pesticide residues in the hive environment. Sci Total Environ. (2021) 805:150292. 10.1016/j.scitotenv.2021.150292 34536857

[10] Wright GA Schiestl FP. The evolution of floral scent: the influence of olfactory learning by insect pollinators on the honest signalling of floral rewards. Funct Ecol. (2009) 23:841–51. 10.1111/j.1365-2435.2009.01627.x

[11] Dötterl S Vereecken NJ. The chemical ecology and evolution of bee-flower interactions: a review and perspectives. Can J Zool. (2010) 88:668–97. 10.1139/Z10-031

[12] Goulson D Chapman JW Hughes WO. Discrimination of unrewarding flowers by bees; direct detection of rewards and use of repellent scent marks. J Insect Behav. (2001) 14:669–78. 10.1023/A:1012231419067

[13] Ma R Villar G Grozinger CM Rangel J. Larval pheromones act as colony-wide regulators of collective foraging behavior in honeybees. Behav Ecol. (2018) 29:1132–41. 10.1093/beheco/ary090

[14] Paoli M Galizia GC. Olfactory coding in honeybees. Cell Tissue Res. (2021) 383:35–58. 10.1007/s00441-020-03385-5 33443623 PMC7873095

[15] Baracchi D Cabirol A Devaud J-M Haase A D'Ettorre P Giurfa M. Pheromone components affect motivation and induce persistent modulation of associative learning and memory in honey bees. Commun Biol. (2020) 3:447. 10.1038/s42003-020-01183-x 32807870 PMC7431541

[16] Sandrock C Tanadini M Tanadini LG Fauser-Misslin A Potts SG Neumann P. Impact of chronic neonicotinoid exposure on honeybee colony performance and queen supersedure. PLoS ONE. (2014) 9:e103592. 10.1371/journal.pone.0103592 25084279 PMC4118897

[17] Fischer J Mueller T Spatz AK Greggers U Gruenewald B Menzel R. Neonicotinoids interfere with specific components of navigation in honeybees. PLoS ONE. (2014) 9:e91364. 10.1371/journal.pone.0091364 24646521 PMC3960126

[18] Andrione M Vallortigara G Antolini R Haase A. Neonicotinoid-induced impairment of odour coding in the honeybee. Sci Rep. (2016) 6:1–9. 10.1038/srep38110 27905515 PMC5131477

[19] Cabirol A Haase A. The neurophysiological bases of the impact of neonicotinoid pesticides on the behaviour of honeybees. Insects. (2019) 10:344. 10.3390/insects10100344 31614974 PMC6835655

[20] Pelosi P. Perireceptor events in olfaction. J Neurobiol. (1996) 30:3–19. 8727979 10.1002/(SICI)1097-4695(199605)30:1<3::AID-NEU2>3.0.CO;2-A

[21] Giacomuzzi V Cappellin L Khomenko I Biasioli F Schütz S Tasin M. Emission of volatile compounds from apple plants infested with *Pandemis heparana* larvae, antennal response of conspecific adults, and preliminary field trial. J Chem Ecol. (2016) 42:1265–80. 10.1007/s10886-016-0794-8 27896554

[22] Frasnelli E Anfora G Trona F Tessarolo F Vallortigara G. Morpho-functional asymmetry of the olfactory receptors of the honeybee (*Apis mellifera*). Behav Brain Res. (2010) 209:221–5. 10.1016/j.bbr.2010.01.046 20138089

[23] Van Den Dool H Kratz PD. A generalization of the retention index system including linear temperature programmed gas-liquid partition chromatography. J Chromatogr. (1963) 11:463–71. 10.1016/s0021-9673(01)80947 14062605

[24] Straub F Orih IJ Kimmich J Ayasse M. Negative effects of the neonicotinoid clothianidin on foraging behavior and antennal sensitivity in two common pollinator species, *Osmia bicornis* and *Bombus terrestris*. Front Ecol Evol. (2021) 9:697355. 10.3389/fevo.2021.697355

[25] R Core Team (2020). R: A language and environment for statistical computing. R Foundation for Statistical Computing. Vienna, Austria. Available online at: https://www.R-project.org/.

[26] Lenth R. EMMEANS: Estimated Marginal Means, Aka Least-Squares Means. R Package Version 1.3.5.1 (2019).

[27] Venables WN Ripley BD. Modern Applied Statistics With S, Fourth edition. Springer, New York. (2002).

[28] Kuznetsova A Brockhoff PB Christensen RHB. “lmerTest package: tests in linear mixed effects models.” *J Stat Softw*. (2017) 82:1–26. 10.18637/jss.v082.i13

[29] Bates D Mächler M Bolker B Walker S. Fitting linear mixed-effects models using lme4. J Stat Softw. (2015) 67:1–48. 10.18637/jss.v067.i01

[30] Wickham H. GGPLOT2: Elegant Graphics for Data Analysis. Springer-Verlag New York (2016).

[31] Hartig F. DHARMa: Residual Diagnostics for Hierarchical (Multi-Level/Mixed) Regression Models. R package version 0.4.1 (2021).

[32] Benjamini Y Hochberg Y. Controlling the false discovery rate: a practical and powerful approach to multiple testing. J R Stat Soc Series B. (1995) 57:289–300. 10.1111/j.2517-6161.1995.tb02031.x

[33] Baraldi R Rapparini F Rossi F Latella A Ciccioli P. Volatile organic compound emissions from flowers of the most occuring and economically important species of fruit trees. Phys Chem Earth *Part B Hydrol Oceans Atmosphere*. (1999) 24:729–32. 10.1016/S1464-1909(99)00073-8

[34] Fraternale D Ricci D Flamini G Giomaro G. Volatiles profile of red apple from Marche Region (Italy). Rec Nat Prod. (2011) 5:202. 25354879 10.5650/jos.ess14088

[35] Cellini A Giacomuzzi V Donati I Farneti B Rodriguez Estrada MT Savioli S. Pathogen-induced changes in floral scent may increase honeybee-mediated dispersal of *Erwinia amylovora*. ISME J. (2019) 13:847–59. 10.1038/s41396-018-0319-2 30504898 PMC6461938

[36] Qin L Wei QP Kang WH Zhang Q Sun J Liu SZ. Comparison of volatile compounds in ‘Fuji'apples in the different regions in China. Food Sci Technol Res. (2017) 23:79–89. 10.3136/fstr.23.79

[37] Li D Han D Zhang H Zhang C Li M Wang Y. Analysis of aroma volatile compounds in fuji apple using spme with different fiber coatings. Agric Biotechnol. (2020) 9:78–89.

[38] Loughrin JN Hamilton-Kemp TR Andersen RA Hildebrand DF. Volatiles from flowers of Nicotiana sylvestris, N. otophora and Malus × domestica: headspace components and day/night changes in their relative concentrations. Phytochemistry. (1990) 29:2473–7. 10.1016/0031-9422(90)85169-G

[39] Buchbauer G Jirovetz L Wasicky M Nikiforov A. Headspace and essential oil analysis of apple flowers. J Agric Food Chem. (1993) 41:116–8. 10.1021/jf00025a025

[40] Rachersberger M. Cordeiro G. D. Schäffler I. Dötterl S. Honeybee pollinators use visual and floral scent cues to find apple (*Malus domestica*) flowers. J Agric Food Chem. (2019). 67:13221–7. 10.1021/acs.jafc.9b06446 31682121

[41] El-Sayed AM Sporle A Colhoun K Furlong J White R Suckling DM. Scents in orchards: floral volatiles of four stone fruit crops and their attractiveness to pollinators. Chemoecology. (2018) 28:39–49. 10.1007/s00049-018-0254-8

[42] Kamdem DP Gruber K Barkman T Gage DA. Characterization of black locust floral fragrance. J Essent Oil Res. (1994) 6:199–200. 10.1080/10412905.1994.9698356

[43] Andersson S Nilsson LA Groth I Bergström G. Floral scents in butterfly-pollinated plants: possible convergence in chemical composition. Bot J Linn Soc. (2002) 140:129–53. 10.1046/j.1095-8339.2002.00068.x

[44] Saroglou V Marin PD Rancic A Veljic M Skaltsa H. Composition and antimicrobial activity of the essential oil of six *Hypericum* species from Serbia. Biochem Syst Ecol. (2007) 35:146–52. 10.1016/j.bse.2006.09.00923416368

[45] Patte F Etcheto M Marfaing P Laffort P. Electroantennogram stimulus-response curves for 59 odourants in the honey bee, *Apis mellifica*. J Insect Physiol. (1989) 35:667–75. 10.1016/0022-1910(89)90086-3

[46] Henning JA Teuber LR. Combined gas chromatography-electroantennogram characterization of alfalfa floral volatiles recognized by honey bees (Hymenoptera: Apidae). J Econ Entomol. (1992) 85:226–32. 10.1093/jee/85.1.226

[47] Ma W Long D Wang Y Li X Huang J Shen J. Electrophysiological and behavioral responses of Asian and European honeybees to pear flower volatiles. J Asia Pac Entomol. (2021) 24:221–8. 10.1016/j.aspen.2020.12.011

[48] Visser JH. Electroantennogram responses of the Colorado beetle, *Leptinotarsa decemlineata*, to plant volatiles. Entomol Exp Appl. (1979) 25:86–97. 10.1111/j.1570-7458.1979.tb02851.x

[49] Chen L Fadamiro HY. Differential electroantennogram response of females and males of two parasitoid species to host-related green leaf volatiles and inducible compounds. Bull Entomol Res. (2007) 97:515–22. 10.1017/S0007485307005172 17916269

[50] Andersson MN Schlyter F Hill SR Dekker T. What reaches the antenna? How to calibrate odor flux and ligand–receptor affinities. Chem Senses. (2012) 37:403–20. 10.1093/chemse/bjs009 22362868

[51] Bortolotti L Costa C. “Chemical communication in the honey bee society”. In: Mucignat-Caretta C editor. Neurobiology of Chemical Communication. Boca Raton, FL: CRC Press/Taylor & Francis (2014). p. 147–210. 24830041

[52] Blum MS Fales HM Tucker KW Collins AM. Chemistry of the sting apparatus of the worker honeybee. J Apic Res. (1978) 17:218–21.

[53] Collins AM Blum MS. Alarm responses caused by newly identified compounds derived from the honeybee sting. J Chem Ecol. (1983) 9:57–65. 10.1007/BF00987770 24408619

[54] Wang Z Tan K. Honey bee alarm pheromone mediates communication in plant–pollinator–predator interactions. Insects. (2019) 10:366. 10.3390/insects10100366 31640201 PMC6835895

[55] Collins AM Blum MS. Bioassay of compounds derived from the honeybee sting. J Chem Ecol. (1982) 8:463–70. 10.1007/BF00987794 24414957

[56] Costa H Talora DC Palma MS Chaud-Netto J. Chemical communication in Apis mellifera: temporal modulation of alarm behaviors. J Venom Anim Toxins. (1996) 2:39–45. 10.1590/S0104-79301996000100005

[57] Wager BR Breed MD. Does honey bee sting alarm pheromone give orientation information to defensive bees? Ann Entomol Soc Am. (2000) 93:1329–32. 10.1603/0013-8746(2000)093[1329:DHBSAP]2.0.CO;2

[58] Free JB Ferguson AW Simpkins JR. Honeybee responses to chemical components from the worker sting apparatus and mandibular glands in field tests. J Apic Res. (1989). 28:7–21. 10.1080/00218839.1989.11100814

[59] Shehata SM Townsend GF Shuel RW. Seasonal physiological changes in queen and worker honeybees. J Apic Res. (1981) 20:69–78. 10.1080/00218839.1981.11100475

[60] Skirkevièius A Skirkevièien Z. New data on annual sensitivity dynamics of pheromonal receptors in worker honeybees (*Apis mellifera* L.). Pheromones. (1999) 6:21–6.

[61] Enjin A. Humidity sensing in insects - from ecology to neural processing. Curr Opin Insect Sci. (2017) 24:1–6. 10.1016/j.cois.2017.08.004 29208217

[62] Yokohari F Tominaga Y Tateda H. Antennal hygroreceptors of the honey bee, *Apis mellifera* L. Cell Tissue Res. (1982) 226:63–73. 10.1007/BF00217082 7127426

[63] Tichy H Kallina W. Sensitivity of honeybee hygroreceptors to slow humidity changes and temporal humidity variation detected in high resolution by mobile measurements. PLoS ONE. (2014) 9:e99032. 10.1371/journal.pone.0099032 24901985 PMC4047084

[64] Tichy H Kallina W. Insect hygroreceptor responses to continuous changes in humidity and air pressure. J Neurophysiol. (2010) 103:3274–86. 10.1152/jn.01043.2009 20375249 PMC3206210

[65] Awad AM Moustafa AF Abdel-Rahman MQ. Influence of different statuses of honey bee queens, *Apis mellifera* L. on the ultrastructure of the flagella on (3-day old) workers. Open Entomol J. (2014) 8:22–36. 10.2174/1874407901408010022

[66] Riveros AJ Gronenberg W. Sensory allometry, foraging task specialization and resource exploitation in honeybees. Behav Ecol Sociobiol. (2010) 64:955–66. 10.1007/s00265-010-0911-6

[67] Gong Y Diao Q. Current knowledge of detoxification mechanisms of xenobiotic in honey bees. Ecotoxicology. (2017) 26:1–12. 10.1007/s10646-016-1742-7 27819118

[68] Siede R Faust L Meixner MD Maus C Grünewald B Büchler R. Performance of honey bee colonies under a long-lasting dietary exposure to sublethal concentrations of the neonicotinoid insecticide Thiacloprid. Pest Manag Sci. (2017) 73:1334–44. 10.1002/ps.4547 28168846 PMC5485166

[69] Paoli M Andrione M Haase A. “Imaging Techniques in Insects,” in Lateralized Brain Functions: Methods in Human and Non-Human Species. Rogers LJ, Vallortigara G, editors. New York, NY: Springer New York (2017).

[70] Decourtye A Devillers J Cluzeau S Charreton M Pham-Delègue MH. Effects of Imidacloprid and Deltamethrin on associative learning in honeybees under semi-field and laboratory conditions. Ecotoxicol Environ Saf. (2004) 57:410–9. 10.1016/j.ecoenv.2003.08.001 15041263

[71] Williamson SM Wright GA. Exposure to multiple cholinergic pesticides impairs olfactory learning and memory in honeybees. J Exp Biol. (2013) 216:1799–807. 10.1242/jeb.083931 23393272 PMC3641805

[72] Williamson SM Baker DD Wright GA. Acute exposure to a sublethal dose of Imidacloprid and Coumaphos enhances olfactory learning and memory in the honeybee *Apis mellifera*. Invert Neurosci. (2013) 13:63–70. 10.1007/s10158-012-0144-7 23160709 PMC3672510

[73] Mengoni Goñalons C Farina WM. Effects of sublethal doses of Imidacloprid on young adult honeybee behaviour. PLoS ONE. (2015) 10:e0140814. 10.1371/journal.pone.0140814 26488410 PMC4619519

[74] Tison L Rößner A Gerschewski S Menzel R. The neonicotinoid clothianidin impairs memory processing in honey bees. Ecotoxicol Environ Saf. (2019) 180:139–45. 10.1016/j.ecoenv.2019.05.007 31082577

[75] Mustard JA Gott A Scott J Chavarria NL Wright GA. Honeybees fail to discriminate floral scents in a complex learning task after consuming a neonicotinoid pesticide. J Exp Biol. (2020) 223:jeb217174. 10.1242/jeb.217174 32029463 PMC7075050

[76] Wright GA Lutmerding A Dudareva N Smith BH. Intensity and the ratios of compounds in the scent of snapdragon flowers affect scent discrimination by honeybees (*Apis mellifera*). J Comp Physiol. (2005) 191:105–14. 10.1007/s00359-004-0576-6 15711966

[77] Sachse S Galizia CG. The coding of odour-intensity in the honeybee antennal lobe: local computation optimizes odour representation. Eur J Neurosci. (2003) 18:2119–32. 10.1046/j.1460-9568.2003.02931.x 14622173

[78] Carlsson MA Hansson BS. Detection and Coding of Flower Volatiles in Nectar-Foraging Insects. In Biology of Floral Scent. Boca Raton, FL: CRC Press (2006). p. 243–62.

[79] Pham-Delegue MH Etievant P Guichard E Masson C. Sunflower volatiles involved in honeybee discrimination among genotypes and flowering stages. J Chem Ecol. (1989) 15:329–43. 10.1007/BF02027794 24271447

[80] Wright GA Skinner BD Smith BH. Ability of honeybee, *Apis mellifera*, to detect and discriminate odors of varieties of canola (*Brassica rapa* and *Brassica napus*) and snapdragon flowers (*Antirrhinum majus*). J Chem Ecol. (2002) 28:721–40. 10.1023/A:1015232608858 12035922

[81] Henry M Beguin M Requier F Rollin O Odoux JF Aupinel P. A common pesticide decreases foraging success and survival in honey bees. Science. (2012) 336:348–50. 10.1126/science.1215039 22461498

[82] Hopwood J Vaughan M Shepherd M Biddinger D Mader E Black SH. Are Neonicotinoids Killing Bees? A Review of Research into the Effects of Neonicotinoid Insecticides on Bees, with Recommendations for Action. Xerces Society for Invertebrate Conservation, USA (2012).

[83] Tan K Chen W Dong S Liu X Wang Y Nieh JC. Imidacloprid alters foraging and decreases bee avoidance of predators. PLoS ONE. (2014) 9:e102725. 10.1371/journal.pone.0102725 25025334 PMC4099376

[84] Tison L Hahn ML Holtz S Rößner A Greggers U Bischoff G Menzel R. Honey bees' behavior is impaired by chronic exposure to the neonicotinoid Thiacloprid in the field. Environ Sci Technol. (2016) 50:7218–27. 10.1021/acs.est.6b02658 27268938

[85] Tosi S Burgio G Nieh JC. A common neonicotinoid pesticide, thiamethoxam, impairs honey bee flight ability. Sci Rep. (2017) 7:1–8. 10.1038/s41598-017-01361-8 28446783 PMC5430654

[86] Wadhams LJ Blight MM Kerguelen V Le Métayer M Marion-Poll F Masson C. Discrimination of oilseed rape volatiles by honey bee: novel combined gas chromatographic-electrophysiological behavioral assay. J Chem Ecol. (1994) 20:3221–31. 10.1007/BF02033722 24241988

[87] de Jong R Pham-Delègue MH. Electroantennogram responses related to olfactory conditioning in the honey bee (*Apis mellifera* ligustica). J Insect Physiol. (1991) 37:319–24. 10.1016/0022-1910(91)90066-9

[88] Sandoz J Pham-Delègue M Renou M Wadhams L. Asymmetrical generalisation between pheromonal and floral odours in appetitive olfactory conditioning of the honey bee (*Apis mellifera* L.). J Comp Physiol A Sensory Neura Behav Physiol. (2001) 187:559–68. 10.1007/s003590100228 11730303

[89] Bhagavan S Smith BH. Olfactory conditioning in the honey bee, *Apis mellifera*: effects of odor intensity. Physiol Behav. (1997) 61:107–17. 10.1016/S0031-9384(96)00357-5 8976540

[90] Bonmatin JM Marchand PA Charvet R Moineau I Bengsch ER Colin ME. Quantification of Imidacloprid uptake in maize crops. J Agric Food Chem. (2005) 53:5336–41. 10.1021/jf0479362 15969515

[91] Krischik VA Landmark AL Heimpel GE. Soil-applied Imidacloprid is translocated to nectar and kills nectar-feeding *Anagyrus pseudococci* (Girault)(Hymenoptera: Encyrtidae). Environ Entomol. (2014) 36:1238–45. 10.1603/0046-225X(2007)36[1238:SIITTN]2.0.CO;2 18284749

[92] Alburaki M Chen D Skinner JA Meikle WG Tarpy DR Adamczyk J. Honey bee survival and pathogen prevalence: from the perspective of landscape and exposure to pesticides. Insects. (2018) 9:65. 10.3390/insects9020065 29899302 PMC6023357

[93] Favaro R Bauer LM Rossi M D'Ambrosio L Bucher E Angeli S. Botanical origin of pesticide residues in pollen loads collected by honeybees during and after apple bloom. Front Physiol. (2019) 10:1069. 10.3389/fphys.2019.01069 31620006 PMC6759928

[94] Dively GP Embrey MS Kamel A Hawthorne DJ Pettis JS. Assessment of chronic sublethal effects of Imidacloprid on honey bee colony health. PLoS ONE. (2015) 10:e0118748. 10.1371/journal.pone.0118748 25786127 PMC4364903

[95] Ramirez-Romero R Chaufaux J Pham-Delègue MH. Effects of Cry1Ab protoxin, deltamethrin and Imidacloprid on the foraging activity and the learning performances of the honeybee *Apis mellifera*, a comparative approach. Apidologie. (2005) 36:601–11. 10.1051/apido:2005039

[96] Meikle WG Adamczyk JJ Weiss M Gregorc A Johnson DR Stewart SD. Sublethal effects of Imidacloprid on honey bee colony growth and activity at three sites in the US. PLoS ONE. (2016) 11:e0168603. 10.1371/journal.pone.0168603 28030617 PMC5193417

[97] Tappert L Pokorny T Hofferberth J Ruther J. Sublethal doses of Imidacloprid disrupt sexual communication and host finding in a parasitoid wasp. Sci Rep. (2017) 7:1–9. 10.1038/srep42756 28198464 PMC5309895

[98] Palmer MJ Moffat C Saranzewa N Harvey J Wright G Connolly CN. Cholinergic pesticides cause mushroom body neuronal inactivation in honeybees. Nat Commun. (2013) 4:1634. 10.1038/ncomms2648 23535655 PMC3621900

[99] Schneider D. Electrophysiological investigation on the antennal receptors of the silk moth during chemical and mechanical stimulation. Experientia. (1957) 13:89–91. 10.1007/BF0216011013414779

[100] Jacob VE. Current source density analysis of electroantennogram recordings: a tool for mapping the olfactory response in an insect antenna. Front Cell Neurosci. (2018) 12:287. 10.3389/fncel.2018.00287 30233325 PMC6135050

[101] Li H Wu F Zhao L Tan J Jiang H Hu F. Neonicotinoid insecticide interact with honeybee odorant-binding protein: implication for olfactory dysfunction. Int J Biol Macromol. (2015) 81:624–30. 10.1016/j.ijbiomac.2015.08.055 26318218

[102] Fent K Schmid M Hettich T Schmid S. The neonicotinoid thiacloprid causes transcriptional alteration of genes associated with mitochondria at environmental concentrations in honey bees. Environ Pollut. (2020) 266:115297. 10.1016/j.envpol.2020.115297 32823041

[103] Christen V Krebs J Bünter I Fent K. Biopesticide spinosad induces transcriptional alterations in genes associated with energy production in honey bees (*Apis mellifera*) at sublethal concentrations. J Hazard Mater. (2019) 378:120736. 10.1016/j.jhazmat.2019.06.013 31202068

[104] Tiraboschi E Leonardelli L Segata G Haase A. Parallel processing of olfactory and mechanosensory information in the honeybee antennal lobe. Front Physiol. (2021) 12:790453. 10.3389/fphys.2021.790453 34950059 PMC8691435

[105] Shearer DA Boch R. 2-Heptanone in the mandibular gland secretion of the honey-bee. Nature. (1965) 206:530. 10.1038/206530a05831852

[106] Nouvian M Reinhard J Giurfa M. The defensive response of the honeybee *Apis mellifera*. J Exp Biol. (2016) 219:3505–17. 10.1242/jeb.143016 27852760

[107] Balderrama N Núñez J Guerrieri F Giurfa M. Different functions of two alarm substances in the honeybee. J Comp Physiol A. (2002) 188:485–91. 10.1007/s00359-002-0321-y 12122467

[108] Papachristoforou A Kagiava A Papaefthimiou C Termentzi A Fokialakis N Skaltsounis A-L. (2012). The bite of the honeybee: 2-heptanone secreted from honeybee mandibles during a bite acts as a local anaesthetic in insects and mammals. PLoS ONE. (2012) 7:e47432. (47432). 10.1371/journal.pone.0047432 23091624 PMC3472974

[109] Slessor KN Winston ML Le Conte Y. Pheromone communication in the honeybee (*Apis mellifera* L.). J Chem Ecol. (2005) 31:2731–45. 10.1007/s10886-005-7623-9 16273438

[110] Slessor KN Kaminski L-A King GGS Borden JH Winston ML. Semiochemical basis of the retinue response to queen honey bees. Nature. (1988) 332:354–6. 10.1038/332354a0

[111] Trhlin M Rajchard J. Chemical communication in the honeybee (*Apis mellifera* L.): a review. Vet Med. (2011) 56:265–73. 10.17221/1543-VETMED

[112] European Food Safety Authority. Conclusion on the peer review of the pesticide risk assessment for bees for the active substance clothianidin. EFSA J. (2013) 11:3066. 10.2903/j.efsa.2013.3066 PMC7009451 32625812

[113] Pistorius J Wehner A Kriszan M Bargen H Knaebe S Klein O. Application of predefined doses of neonicotinoid containing dusts in field trials and acute effects on honey bees. Bull Insectology. (2015) 68:161–72.

[114] Bortolotti L Sabatini AG Mutinelli F Astuti M Lavazza A Piro R. Spring honey bee losses in Italy. Julius-Kühn-Archiv. (2009) 423:148–52.27182604

[115] Barbieri RF Lester PJ Miller AS Ryan KG. A neurotoxic pesticide changes the outcome of aggressive interactions between native and invasive ants. Proc R Soc B Biol Sci. (2013) 280:20132157. 10.1098/rspb.2013.2157 24266038 PMC3813335

[116] Sakofski F Koeniger N Fuchs S. Seasonality of honey bee colony invasion by *Varroa jacobsoni* Oud. Apidologie. (1990) 21:547–50. 10.1051/apido:19900608

[117] Frey E Rosenkranz P. Autumn invasion rates of *Varroa destructor* (Mesostigmata: Varroidae) into honey bee (Hymenoptera: Apidae) colonies and the resulting increase in mite populations. J Econ Entomol. (2014) 107:508–15. 10.1603/EC13381 24772528

[118] Williams GR Troxler A Retschnig G Roth K Yañez O Shutler D. Neonicotinoid pesticides severely affect honey bee queens. Sci Rep. (2015) 5:1–8. 10.1038/srep14621 26459072 PMC4602226

[119] Wu-Smart J Spivak M. Sub-lethal effects of dietary neonicotinoid insecticide exposure on honey bee queen fecundity and colony development. Sci Rep. (2016) 6:1–11. 10.1038/srep32108 27562025 PMC4999797

[120] Brandt A Grikscheit K Siede R Grosse R Meixner MD Büchler R. Immunosuppression in honeybee queens by the neonicotinoids Thiacloprid and Clothianidin. Sci Rep. (2017) 7:1–12. 10.1038/s41598-017-04734-1 28680118 PMC5498664

[121] Dussaubat C Maisonnasse A Crauser D Tchamitchian S Bonnet M Cousin M. Combined neonicotinoid pesticide and parasite stress alter honeybee queens' physiology and survival. Sci Rep. (2016) 6:1–7. 10.1038/srep31430 27578396 PMC5005999

[122] Vergara-Amado J Manzi C Franco LM Contecha SC Marquez SJ Solano-Iguaran JJ. Effects of residual doses of neonicotinoid (Imidacloprid) on metabolic rate of queen honey bees *Apis mellifera* (Hymenoptera: Apidae). Apidologie. (2020) 51:1091–9. 10.1007/s13592-020-00787-w

[123] Caselli A Favaro R Petacchi R Angeli S. Infestation of the gall midge Dasineura oleae provides first evidence of induced plant volatiles in olive leaves. Bull. Entomol Res. (2021) 1–13. 10.1017/S0007485321001000 34930508

[124] Maisonnasse A Lenoir JC Beslay D Crauser D Le Conte Y. *E*-β-ocimene, a volatile brood pheromone involved in social regulation in the honey bee colony (*Apis mellifera*). PLoS ONE. (2010) 5:e13531. 10.1371/journal.pone.0013531 21042405 PMC2958837

