# Supplementary material for: Impact of Chronic Exposure to Two Neonicotinoids on Honey Bee Antennal Responses to Flower Volatiles and Pheromonal Compounds
Source: Front Insect Sci. 2022 Apr 18;2:821145. doi: 10.3389/finsc.2022.821145 (PMC10926470; doi:10.3389/finsc.2022.821145)
Supplement: Supplementary file 1 [file Table_1.docx]

# Supplementary Material

**Table S1**: **Antennal responses to flower volatiles.** EAG signals normalized to the positive control stimulus (citral 10^-2^) as a function of odour compound, season (bees collected in September (*N* = 72) or April (*N* = 36)) and treatment (Imidacloprid 50 ppb or Thiacloprid 4500 ppb in sugar syrup, Control: pure syrup).

| \| **Compound** \| **Season** \| **Treatment** \| **Mean ± SD [%] as a function of concentration** \| \| \| \| \| \| --- \| --- \| --- \| --- \| --- \| --- \| --- \| --- \| \|  \|  \|  \| 10^-6^ \| 10^-5^ \| 10^-4^ \| 10^-3^ \| 10^-2^ \| \| Linalool \| Autumn \| Control \| 3 ± 2.6 \| 4 ± 2.1 \| 8 ± 3.9 \| 29 ± 5.8 \| 70.5 ± 10.1 \| \| Imidacloprid \| 4.5 ± 5.1 \| 5 ± 3.1 \| 8.9 ± 5.5 \| 29.5 ± 5.8 \| 68.9 ± 11.3 \| \| Thiacloprid \| 2.7 ± 1.4 \| 3.2 ± 2.4 \| 7.6 ± 3 \| 28.1 ± 6.6 \| 71.5 ± 10.1 \| \| Spring \| Control \| 5.6 ± 0.4 \| 5.12 \| 4 ± 2 \| 21.9 ± 4.6 \| 70.4 ± 9.3 \| \| Imidacloprid \| 2.5 ± 2.8 \| 2 ± 1.8 \| 3.9 ± 3.3 \| 25.2 ± 4.9 \| 72.7 ± 9.3 \| \| Thiacloprid \| 1.7 ± 0.1 \| 0.6 ± 0.5 \| 4.2 ± 3 \| 24.2 ± 5.3 \| 72.8 ± 7 \| \| Hexyl acetate \| Autumn \| Control \| 3.8 ± 2.9 \| 2.6 ± 2.7 \| 7.1 ± 5.5 \| 40.2 ± 17.5 \| 117.9 ± 13.3 \| \| Imidacloprid \| 3.3 ± 1.9 \| 3.8 ± 3 \| 6.3 ± 3.7 \| 38.4 ± 8.7 \| 118 ± 15 \| \| Thiacloprid \| 2.2 ± 0.9 \| 5.7 ± 3.2 \| 4.1 ± 2.7 \| 38.9 ± 5.6 \| 119.7 ± 13.9 \| \| Spring \| Control \| 1.6 ± 2.2 \| 2.3 ± 2.4 \| 2.9 ± 2.5 \| 27.4 ± 6.5 \| 101.7 ± 13.1 \| \| Imidacloprid \| 2.4 ± 1.2 \| 2.2 ± 2.2 \| 4 ± 1.3 \| 28.2 ± 4 \| 106.6 ± 12.8 \| \| Thiacloprid \| 1.1 ± 1 \| 2.1 \| 1.8 ± 2 \| 27.4 ± 4 \| 104.1 ± 14.9 \| \| *(Z)*-3-hexen-1-ol \| Autumn \| Control \| 3.7 ± 3.7 \| 3.8 ± 2.6 \| 8.5 ± 4.2 \| 37 ± 17.2 \| 152.4 ± 25.8 \| \| Imidacloprid \| 5 ± 4.5 \| 5.1 ± 3.7 \| 10.7 ± 8.2 \| 37.6 ± 14 \| 164.3 ± 35.7 \| \| Thiacloprid \| 3.1 ± 2.2 \| 2.2 ± 2.2 \| 7.6 ± 3.8 \| 41.4 ± 9.6 \| 162 ± 19 \| \| Spring \| Control \| 5.2 \| 2.5 ± 2 \| 5.4 ± 4 \| 37.9 ± 10.3 \| 142.1 ± 38.2 \| \| Imidacloprid \| - \| 1.4 ± 1.3 \| 5.2 ± 2.3 \| 38.1 ± 8.6 \| 147.6 ± 25.4 \| \| Thiacloprid \| - \| 1.1 ± 0.8 \| 5.9 ± 4.1 \| 40.6 ± 11.2 \| 156.3 ± 30.9 \| \| Methyl salicylate \| Autumn \| Control \| 4.6 ± 3.9 \| 4 ± 3.2 \| 7.4 ± 3.3 \| 18 ± 4.8 \| 59.3 ± 9.3 \| \| Imidacloprid \| 4.4 ± 5 \| 4 ± 2.3 \| 7.6 ± 3.9 \| 19.6 ± 9.5 \| 58.4 ± 11.6 \| \| Thiacloprid \| 3 ± 2 \| 4.3 ± 3.1 \| 7 ± 2.7 \| 18.3 ± 4.8 \| 60 ± 6.2 \| \| Spring \| Control \| 3.3 ± 2.8 \| 2.2 ± 2.7 \| 7.2 ± 4.8 \| 19 ± 2.5 \| 69.3 ± 7.4 \| \| Imidacloprid \| 3.7 ± 5.2 \| 2.7 ± 1.6 \| 4.6 ± 2.6 \| 16.3 ± 4.1 \| 66.6 ± 7.8 \| \| Thiacloprid \| 1.8 ± 1.1 \| 2.3 ± 2.1 \| 4.7 ± 2.6 \| 14.1 ± 3.7 \| 67.9 ± 8.6 \| \| *(E)*-β-ocimene \| Autumn \| Control \| 4.1 ± 3.4 \| 3.8 ± 2.8 \| 6.3 ± 4 \| 9.2 ± 4.1 \| 26.7 ± 5.9 \| \| Imidacloprid \| 5.3 ± 5.2 \| 5 ± 4.5 \| 7.3 ± 3.9 \| 10.8 ± 5.4 \| 27.1 ± 7 \| \| Thiacloprid \| 2 ± 1.2 \| 2.4 ± 1.5 \| 5.1 ± 3.2 \| 7.6 ± 3.8 \| 22.1 ± 4.9 \| \| Spring \| Control \| - \| 1.1 \| 1.46 \| 4.6 ± 2.7 \| 19.1 ± 2.9 \| \| Imidacloprid \| - \| 1.9 \| 1.7 ± 1.1 \| 5.5 ± 2.8 \| 22 ± 3.9 \| \| Thiacloprid \| 1.6 \| - \| 3 ± 1.4 \| 5.7 ± 4.6 \| 21.1 ± 5.3 \| \| 1-hexanol \| Autumn \| Control \| 4.7 ± 4.1 \| 6.1 ± 6.6 \| 15.3 ± 4.5 \| 55 ± 15.1 \| 203.8 ± 25.5 \| \| Imidacloprid \| 5.1 ± 5.7 \| 5.5 ± 4.6 \| 17.4 ± 9.1 \| 60.4 ± 19.8 \| 207.9 ± 33.1 \| \| Thiacloprid \| 3.7 ± 3.2 \| 4.3 ± 2.3 \| 17.7 ± 6 \| 74.3 ± 11.4 \| 213.2 ± 25.6 \| \| Spring \| Control \| 2.5 ± 1.7 \| 1.9 ± 3.3 \| 12.5 ± 3.8 \| 53.2 ± 12.4 \| 181.1 ± 17.7 \| \| Imidacloprid \| 3.3 ± 1.4 \| 3.6 ± 2.9 \| 11 ± 4.6 \| 54.5 ± 8.8 \| 179.7 ± 24 \| \| Thiacloprid \| 1.5 ± 0.5 \| 2.1 ± 1.2 \| 14.2 ± 4.1 \| 59 ± 15.3 \| 191.5 ± 29 \| \| Benzyl alcohol \| Autumn \| Control \| 6.2 ± 5.7 \| 3.8 ± 2.8 \| 6.1 ± 4.1 \| 17 ± 6.4 \| 48 ± 15 \| \| Imidacloprid \| 5.1 ± 3.9 \| 5.2 ± 3.5 \| 6.9 ± 5.3 \| 17.2 ± 6.4 \| 53 ± 24.5 \| \| Thiacloprid \| 4.9 ± 3.5 \| 4.5 ± 2.4 \| 6.5 ± 4.1 \| 31 ± 9.2 \| 73.9 ± 16.7 \| \| Spring \| Control \| 3.8 ± 2.5 \| 2 ± 2.2 \| 4.2 ± 2.9 \| 32.1 ± 8.5 \| 95.4 ± 16.7 \| \| Imidacloprid \| 3.1 ± 0.1 \| 0.9 ± 1.1 \| 4.1 ± 2.4 \| 30.3 ± 7.9 \| 90.6 ± 11.4 \| \| Thiacloprid \| 0.8 ± 0.8 \| 3.8 ± 2.8 \| 3.7 ± 2 \| 34.3 ± 6.3 \| 100.7 ± 18 \| \| α-terpineol \| Autumn \| Control \| 11.4 ± 28.7 \| 4.4 ± 4.8 \| 10.2 ± 5.8 \| 24 ± 6.8 \| 92.8 ± 16.9 \| \| Imidacloprid \| 4.5 ± 5.4 \| 5.4 ± 5.5 \| 11.2 ± 10.1 \| 27.7 ± 13.4 \| 99.3 ± 18.6 \| \| Thiacloprid \| 2.6 ± 2.2 \| 3.2 ± 2.7 \| 3.6 ± 2.8 \| 19.5 ± 5.9 \| 85.5 ± 13.6 \| \| Spring \| Control \| 3.1 \| 2.1 \| 0.3 \| 14.1 ± 3.3 \| 66.5 ± 9.7 \| \| Imidacloprid \| - \| - \| 4 ± 0.1 \| 13.9 ± 2.7 \| 67 ± 4.7 \| \| Thiacloprid \| - \| - \| 1.4 ± 1.3 \| 13.5 ± 3.4 \| 68.3 ± 7.6 \| \| Benzyl acetate \| Autumn \| Control \| 6.6 ± 7 \| 5 ± 4.2 \| 14.9 ± 6 \| 34.3 ± 7.5 \| 92.4 ± 17.4 \| \| Imidacloprid \| 6.8 ± 4.6 \| 7.2 ± 5.2 \| 18.3 ± 13 \| 35.3 ± 9 \| 99 ± 16.5 \| \| Thiacloprid \| 2.4 ± 1.7 \| 1.8 ± 1.7 \| 8.8 ± 4.1 \| 35.4 ± 7 \| 89.5 ± 11.5 \| \| Spring \| Control \| 0.7 ± 0.2 \| - \| 4 ± 2.5 \| 28.3 ± 5.3 \| 85.8 ± 12.3 \| \| Imidacloprid \| 3.6 \| 3.6 ± 1.6 \| 5.6 ± 3.6 \| 31.1 ± 6.5 \| 92 ± 9.8 \| \| Thiacloprid \| 2 ± 1.3 \| 2 ± 1.1 \| 4.5 ± 1.9 \| 30 ± 6.1 \| 88.6 ± 11.7 \| \| 2-heptanone \| Autumn \| Control \| 5.5 ± 5.1 \| 8.7 ± 7.7 \| 18.6 ± 5.5 \| 55 ± 10.2 \| 140 ± 39 \| \| Imidacloprid \| 5.4 ± 5.2 \| 9.2 ± 7 \| 21.4 ± 9.4 \| 59.5 ± 13.5 \| 145 ± 42.1 \| \| Thiacloprid \| 3 ± 2.7 \| 5.2 ± 2.9 \| 19.4 ± 4 \| 69.2 ± 8.8 \| 184.3 ± 25.4 \| \| Spring \| Control \| - \| 0.3 ± 0.1 \| 7 ± 2.9 \| 44.8 ± 9.3 \| 150.6 ± 17.7 \| \| Imidacloprid \| - \| 0.5 ± 0.7 \| 9.5 ± 3.2 \| 47.8 ± 8.1 \| 160.4 ± 17.2 \| \| Thiacloprid \| - \| 1.6 v 1.8 \| 10 ± 3.7 \| 48.5 ± 10 \| 167.9 ± 29.4 \| |
| --- | --- | --- | --- | --- | --- | --- | --- | --- | --- | --- | --- | --- | --- | --- | --- | --- | --- | --- | --- | --- | --- | --- | --- | --- | --- | --- | --- | --- | --- | --- | --- | --- | --- | --- | --- | --- | --- | --- | --- | --- | --- | --- | --- | --- | --- | --- | --- | --- | --- | --- | --- | --- | --- | --- | --- | --- | --- | --- | --- | --- | --- | --- | --- | --- | --- | --- | --- | --- | --- | --- | --- | --- | --- | --- | --- | --- | --- | --- | --- | --- | --- | --- | --- | --- | --- | --- | --- | --- | --- | --- | --- | --- | --- | --- | --- | --- | --- | --- | --- | --- | --- | --- | --- | --- | --- | --- | --- | --- | --- | --- | --- | --- | --- | --- | --- | --- | --- | --- | --- | --- | --- | --- | --- | --- | --- | --- | --- | --- | --- | --- | --- | --- | --- | --- | --- | --- | --- | --- | --- | --- | --- | --- | --- | --- | --- | --- | --- | --- | --- | --- | --- | --- | --- | --- | --- | --- | --- | --- | --- | --- | --- | --- | --- | --- | --- | --- | --- | --- | --- | --- | --- | --- | --- | --- | --- | --- | --- | --- | --- | --- | --- | --- | --- | --- | --- | --- | --- | --- | --- | --- | --- | --- | --- | --- | --- | --- | --- | --- | --- | --- | --- | --- | --- | --- | --- | --- | --- | --- | --- | --- | --- | --- | --- | --- | --- | --- | --- | --- | --- | --- | --- | --- | --- | --- | --- | --- | --- | --- | --- | --- | --- | --- | --- | --- | --- | --- | --- | --- | --- | --- | --- | --- | --- | --- | --- | --- | --- | --- | --- | --- | --- | --- | --- | --- | --- | --- | --- | --- | --- | --- | --- | --- | --- | --- | --- | --- | --- | --- | --- | --- | --- | --- | --- | --- | --- | --- | --- | --- | --- | --- | --- | --- | --- | --- | --- | --- | --- | --- | --- | --- | --- | --- | --- | --- | --- | --- | --- | --- | --- | --- | --- | --- | --- | --- | --- | --- | --- | --- | --- | --- | --- | --- | --- | --- | --- | --- | --- | --- | --- | --- | --- | --- | --- | --- | --- | --- | --- | --- | --- | --- | --- | --- | --- | --- | --- | --- | --- | --- | --- | --- | --- | --- | --- | --- | --- | --- | --- | --- | --- | --- | --- | --- | --- | --- | --- | --- | --- | --- | --- | --- | --- | --- | --- | --- | --- | --- | --- | --- | --- | --- | --- | --- | --- | --- | --- | --- | --- | --- | --- | --- | --- | --- | --- | --- | --- | --- | --- | --- | --- | --- | --- | --- | --- | --- | --- | --- | --- | --- | --- | --- | --- | --- | --- | --- | --- | --- |
