# Supplementary material for: Impact of Chronic Exposure to Two Neonicotinoids on Honey Bee Antennal Responses to Flower Volatiles and Pheromonal Compounds
Source: Front Insect Sci. 2022 Apr 18;2:821145. doi: 10.3389/finsc.2022.821145 (PMC10926470; doi:10.3389/finsc.2022.821145)
Supplement: Supplementary file 2 [file Table_2.DOCX]

# Supplementary Material

| **Table S2: Antennal responses to the control stimuli**. EAG signals (mV) of the antennal responses of bees to the positive control (Citral 10^-2^) and the negative controls (empty Pasteur pipette, Pasteur pipette with paraffin oil) as a function of season (bees collected in September (*N* = 72) or April (*N* =3 6)), treatment (Imidacloprid 50 ppb or Thiacloprid 4500 ppb in sugar syrup, Control: pure syrup) and time of the recording sequence. |
| --- |
| \|  \| **Season** \| **Treatment** \| **Stimulus** \| **Time of recording** \| \| \| \| --- \| --- \| --- \| --- \| --- \| --- \| --- \| \|  \|  \|  \|  \| **Start** \| **Middle** \| **End** \| \| \| Positive Control \| Autumn \| Control \| Citral 10^-2^ \| -0.649 ± 0.117 \| -0.626 ± 0.125 \| -0.589 ± 0.139 \| \| \| Imidacloprid \| Citral 10^-2^ \| -0.712 ± 0.157 \| -0.632 ± 0.153 \| -0.531 ± 0.135 \| \| \| Thiacloprid \| Citral 10^-2^ \| -0.623 ± 0.106 \| -0.532 ± 0.109 \| -0.444 ± 0.118 \| \| \| Spring \| Control \| Citral 10^-2^ \| -0.634 ± 0.122 \| -0.641 ± 0.126 \| -0.62 ± 0.12 \| \| \| Imidacloprid \| Citral 10^-2^ \| -0.697 ± 0.147 \| -0.665 ± 0.108 \| -0.66 ± 0.127 \| \| \| Thiacloprid \| Citral 10^-2^ \| -0.651 ± 0.132 \| -0.643 ± 0.136 \| -0.622 ± 0.125 \| \| \| Negative Control \| Autumn \| Control \| Empty \| -0.083 ± 0.028 \|  \| -0.092 ± 0.026 \| \| \| Control \| Paraffin \| -0.076 ± 0.026 \|  \| -0.08 ± 0.025 \| \| \| Imidacloprid \| Empty \| -0.099 ± 0.037 \|  \| -0.094 ± 0.028 \| \| \| Imidacloprid \| Paraffin \| -0.093 ± 0.037 \|  \| -0.084 ± 0.031 \| \| \| Thiacloprid \| Empty \| -0.085 ± 0.018 \|  \| -0.08 ± 0.025 \| \| \|  \| Thiacloprid \| Paraffin \| -0.086 ± 0.017 \|  \| -0.089 ± 0.03 \| \| \| Spring \| Control \| Empty \| -0.098 ± 0.043 \|  \| -0.121 ± 0.026 \| \| \| Control \| Paraffin \| -0.094 ± 0.047 \|  \| -0.131 ± 0.026 \| \| \| Imidacloprid \| Empty \| -0.098 ± 0.042 \|  \| -0.119 ± 0.023 \| \| \| Imidacloprid \| Paraffin \| -0.108 ± 0.036 \|  \| -0.128 ± 0.018 \| \| \| Thiacloprid \| Empty \| -0.097 ± 0.033 \|  \| -0.116 ± 0.032 \| \| \| Thiacloprid \| Paraffin \| -0.095 ± 0.03 \|  \| -0.119 ± 0.03 \| \| |
